# Identification of Novel Key Genes and Pathways in Multiple Sclerosis Based on Weighted Gene Coexpression Network Analysis and Long Noncoding RNA-Associated Competing Endogenous RNA Network

**DOI:** 10.1155/2022/9328160

**Published:** 2022-03-02

**Authors:** Yuehan Hao, Miao He, Yu Fu, Chenyang Zhao, Shuang Xiong, Xiaoxue Xu

**Affiliations:** ^1^Department of Neurology, The First Hospital of China Medical University, Shenyang, China; ^2^Department of Pharmaceutical Toxicology, School of Pharmacy, China Medical University, Shenyang, China; ^3^Liaoning Academy of Analytic Science, Construction Engineering Center of Important Technology Innovation and Research and Development Base in Liaoning Province, Shenyang, China

## Abstract

**Objective:**

Multiple sclerosis (MS) is an autoimmune disease of the central nervous system characterized by chronic inflammation and demyelination. This study is aimed at identifying crucial genes and molecular pathways involved in MS pathogenesis.

**Methods:**

Raw data in GSE52139 were collected from the Gene Expression Omnibus. The top 50% expression variants were subjected to weighted gene coexpression network analysis (WGCNA), and the key module associated with MS occurrence was identified. A long noncoding RNA- (lncRNA-) associated competing endogenous RNA (ceRNA) network was constructed in the key module. The hub gene candidates were subsequently verified in an individual database.

**Results:**

Of the 18 modules obtained, the cyan module was designated as the key module. The established ceRNA network was composed of seven lncRNAs, 45 mRNAs, and 21 microRNAs (miRNAs), and the *FAM13A-AS1* was the lncRNA with the highest centrality. Functional assessments indicated that the genes in the cyan module primarily gathered in ribosome-related functional terms. Interestingly, the targeted mRNAs of the ceRNA network enriched in diverse categories. Moreover, highly expressed *CYBRD1*, *GNG12*, and *SMAD1*, which were identified as hub genes, may be associated with “valine leucine and isoleucine degradation,” “base excision repair,” and “fatty acid metabolism,” respectively, according to the results of single gene-based genomes and gene set enrichment analysis (GSEA).

**Conclusions:**

Combined with the WGCNA and ceRNA network, our findings provide novel insights into the pathogenesis of MS. The hub genes discovered herein might also serve as novel biomarkers that correlate with the development and management of MS.

## 1. Introduction

The impairment of the immune response leads to the production of aberrant autoantibodies, which damage the immunological tolerance to self-antigens, contributing to several autoimmune disorders (ADs) [1]. Multiple sclerosis (MS) is the most prevalent AD, and patients with MS exhibit immune-mediated inflammation and neuronal sheath loss in the central nervous system. The onset of the disease is characterized by brain and spinal cord atrophy, which advances over the course of the disease. Demyelination blocks nerve conduction and results in progressive neurodegeneration [2–4]. Particularly, spinal cord involvement turns MS into a frequent determinant of neurological disability globally. Although several treatments have been applied to mitigate MS symptoms by modulating or suppressing the immune system, the deficiency of curative strategies remains an important issue that must be urgently solved. A comprehensive understanding of the molecular pathways and hub genes involved will be crucial for developing promising MS therapies.

Long noncoding RNAs (lncRNAs) are a class of noncoding RNAs (ncRNAs) with lengths > 200 nucleotides. lncRNAs can serve as molecular sponges, sequestering DNA, RNA, or protein to regulate gene expression. Dysregulated lncRNAs might contribute to malfunctioning microRNAs (miRNAs) and an abnormal transcription of their mRNA targets, eventually having physiological and pathological implications for patients. Cumulative evidence has confirmed the wide distribution of lncRNAs in diverse immune cells, including T cells, macrophages, and monocytes. Therefore, lncRNA has garnered increasing attention in MS pathogenicity due to its involvement in modulating immune cell behavior and immune system maturation [5, 6]. The aberrant levels of lncRNAs *PINK1-AS*, *LNC-MKI67IP*, and *HNF1A-AS1* were discovered in patients with MS [7, 8]. *GAS5* expression was positively associated with the expanded disability status scale scores of patients with relapsing-remitting MS [9]. The lncRNA, *PVT1*, was found to reduce the pathogenic inflammatory responses in the MS rodent model, suggesting its potential as a therapeutic target [10]. Nevertheless, the precise role of lncRNAs in MS occurrence and development is still unclear. Apart from its complicated mechanism, the absence of a comprehensive lncRNA expression profile creates a huge knowledge gap as to the lncRNA-miRNA-mRNA regulatory network. Investigating how lncRNAs participate in MS pathogenesis is conducible for the diagnosis, prognosis, and treatment of this disease [5, 11].

The current study is designed to uncover the crucial genes and to explore potential molecular pathways involved in MS. We conducted a bioinformatics analysis by combined utilization of several approaches. Weighted gene coexpression network analysis (WGCNA) was first applied to identify the key module highly related to MS occurrence. We then constructed an lncRNA-associated competing endogenous RNA (ceRNA) network based on the genes in the key module only. The hub genes were eventually recognized on the basis of these analyses, and their potential functions were evaluated through functional enrichment assessments. Our findings are expected to provide novel insights into the pathogenesis and progression of MS.

## 2. Materials and Methods

### 2.1. Raw Data Collection and Data Preprocessing

**Figure 1:**
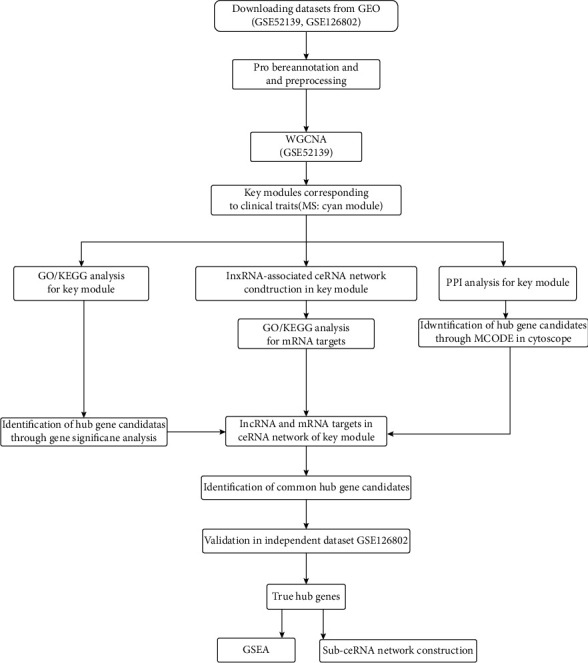
Summary of the analysis pipeline for the study. GSE52139 and GSE126802 were downloaded from the GEO database. After probe reannotation and data preprocessing, weighted gene coexpression network analysis (WGCNA) was performed using GSE52139 to figure out the key module related to MS occurrence. Competing endogenous RNA (ceRNA) network and protein-protein interaction (PPI) network were then constructed in the key module. The hub gene candidates were obtained through intersecting the key genes in WGCNA, ceRNA, and protein-protein interaction (PPI). These candidates were finally validated in GSE126802 to obtain hub genes. Genomes and gene set enrichment analysis (GSEA) were utilized to investigate the potential function of the hub genes.

The total RNA-sequencing datasets of two gene expression profiles (GSE52139 and GSE126802) were obtained from the Gene Expression Omnibus (GEO) database (https://www.ncbi.nlm.nih.gov/gds/). GSE52139 was sequenced using the GPL570 platform (Affymetrix Human Genome U133 Plus 2.0 (HG-U133_Plus_2) Array). GSE52139 includes eight spinal cord samples from patients with MS. The periplaque demyelinating lesions of these patients were compared with the normal-appearing spinal white matter collected from the spinal cord of the same patient (8 periplaque samples vs. 8 normal white matter). For validation, the GSE126802 dataset was assessed using GPL13497 (Agilent-026652 Whole Human Genome Microarray 4 × 44 K v2). The GSE126802 dataset contains normal-appearing subcortical white matter from 18 MS donors and 9 healthy donors. The series matrix file and the annotation table of the platform were downloaded. For the two datasets, the mRNA and lncRNA expression information was obtained by reannotating the gene symbols based on the Encyclopedia of DNA Elements (ENCODE) using ENSEMBL, as described in previous studies [12]. After correcting for the background, all data were preprocessed using the “Impute” package in “R” to average the values of multiple probes for the same gene.  Figure 1 demonstrates the complete workflow of the current project.

### 2.2. Coexpression Network Analysis Using WGCNA

To determine the key groups of genes that are highly associated with MS occurrence, WGCNA was carried out using the “WGCNA” package in “R.” After log2-transformation, a total of 8,819 genes exhibiting the highest (top 50%) variation in expression levels in the GSE52139 dataset were filtered out using the “Limma” package [13] to create a coexpression network [14]. When the degree of independence was 0.85, the lowest power value was selected as the soft-thresholding parameter to ensure a relatively high scale-free network. To acquire the coexpressed gene modules, the weighted adjacency matrix was changed to a topological matrix via topological overlap measure (TOM). According to TOM dissimilarity, the genes with similar expression profiles were categorized into the same modules, and a clustering dendrogram was constructed using linkage hierarchical clustering. In the present study, the minimum gene group size was set to 80, and the threshold used for merging similar modules was set to 0.25. All modules were summarized based on the module eigengenes (MEs), which are the principal components of each module that represent the expression profile of all genes in the module. For each module, the module membership (MM) was defined as the correlation between the ME and the gene expression profile, while gene significance (GS) was defined as the absolute value of the correlation between the gene and the clinical trait. Finally, module significance and the average absolute GS were computed to assess the correlation of the overall module genes with the clinical trait and to identify the key module that is correlated with MS occurrence.

### 2.3. Functional Assessments

Database for Annotation, Visualization, and Integration Discovery (DAVID; https://david.ncifcrf.gov) was used for Gene Ontology (GO) enrichment and the Kyoto Encyclopedia of Genes and Genomes (KEGG) pathway analyses of the genes of interest. Statistical significance was set to *P* < 0.05. The top 10 terms of each category of GO analysis and KEGG pathways were visualized using the “Gobarplot” and “Ggplot2” packages.

### 2.4. Protein-Protein Interaction (PPI) Analysis

The PPI network of genes in the key module was analyzed using the Search Tool for the Retrieval of Interacting Genes (STRING) website (https://string-db.org). The plug-in Molecular Complex Detection (MCODE) of Cytoscape [15] was utilized to check the clusters and visualize the protein interactions. The criteria used for selection were as follows: MCODE score > 5, degree cutoff = 2, maximum depth = 100, *k*‐core = 2, and node score cutoff = 0.2. The potential key genes in the cluster with the highest MCODE score were retrieved.

### 2.5. Potential ceRNA Network Construction in the Key Module

The lncRNAs in the key module were collected to predict the targeted miRNAs using the miRcode database (http://www.mircode.org/). The putative miRNA-mRNA interactions were determined using miRTarBase (http://mirtarbase.mbc.nctu.edu.tw/), miRDB (http://www.mirdb.org/), and TargetScan (http://www.targetscan.org/). The genes that appeared in all three databases were cross-matched with the mRNAs in the key module to acquire the final mRNA targets. The lncRNA-miRNA-mRNA ceRNA network was constructed and visualized using Cytoscape (version 3.7.1). GO enrichment and KEGG pathway analyses of the targeted mRNAs in the ceRNA network were conducted using DAVID, as previously described. The results were visualized using the “GOplot” and “Ggplot2” packages.

### 2.6. Hub Gene Identification and Validation

The genes with higher relevance were filtered according to the module connectivity (measured by MM ≥ median) and the clinical trait relationships (measured by GS ≥ median) [16]. Genes in the key module with an MCODE score greater than 0.4 were collected. These genes and lncRNAs, together with the predicted mRNA targets in the constructed ceRNA network, were employed in a Venn diagram analysis (http://bioinformatics.psb.ugent.be/webtools/Venn/) to identify the hub candidates. As an independent dataset, GSE126802 was employed to verify the true hub genes in MS. After preprocessing, the raw data were extracted, and unpaired Student's *t*-test (two-tailed) was used to evaluate the significant differences between patients with MS and healthy individuals. The diagnostic accuracy of these genes was evaluated using the area under the curve (AUC) of the receiver operating characteristic (ROC) curve using GraphPad Prism 5.0. The significantly altered genes (*P* < 0.05) with an AUC > 0.70 (*P* < 0.05) were identified as the true hub genes.

### 2.7. Genomes and Gene Set Enrichment Analysis (GSEA)

GSEA for the hub genes was conducted using the GSEA 4.1.0 software. The periplaque samples were divided into two groups according to the median expression value of each gene; thus, the samples with an expression level greater than the median were assigned to the high expression group, while those with an expression lower than the median were assigned to the low expression group. The gene set database used was “c2.cp.kegg.v7.4.symbols.gmt.” Gene set permutations for each analysis were executed 1,000 times. An absolute value of the standardized enrichment score > 1 and a nominal *P* value < 0.05 were considered statistically significant.

## 3. Results

### 3.1. Coexpression Network Constructed Using WGCNA

**Figure 2:**
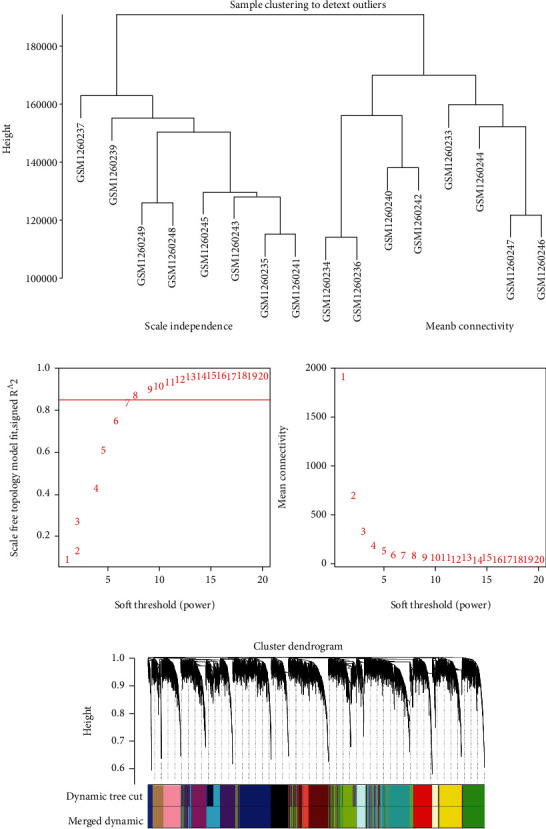
Weighted gene coexpression network analysis (WGCNA). (a) Clustering dendrograms of samples. All 16 samples were divided into two groups. The samples from healthy donors were primarily grouped as one cluster. (b) Determination of the soft-thresholding power (*β*) in WGCNA. A power of *β* = 8 was selected as the soft threshold. (c) Gene clustering tree built using hierarchical clustering of adjacency-based dissimilarity. There are 18 coexpression clusters with the corresponding color assignments. Each branch represents a single gene. The height indicates the Euclidean distance.

**Figure 3:**
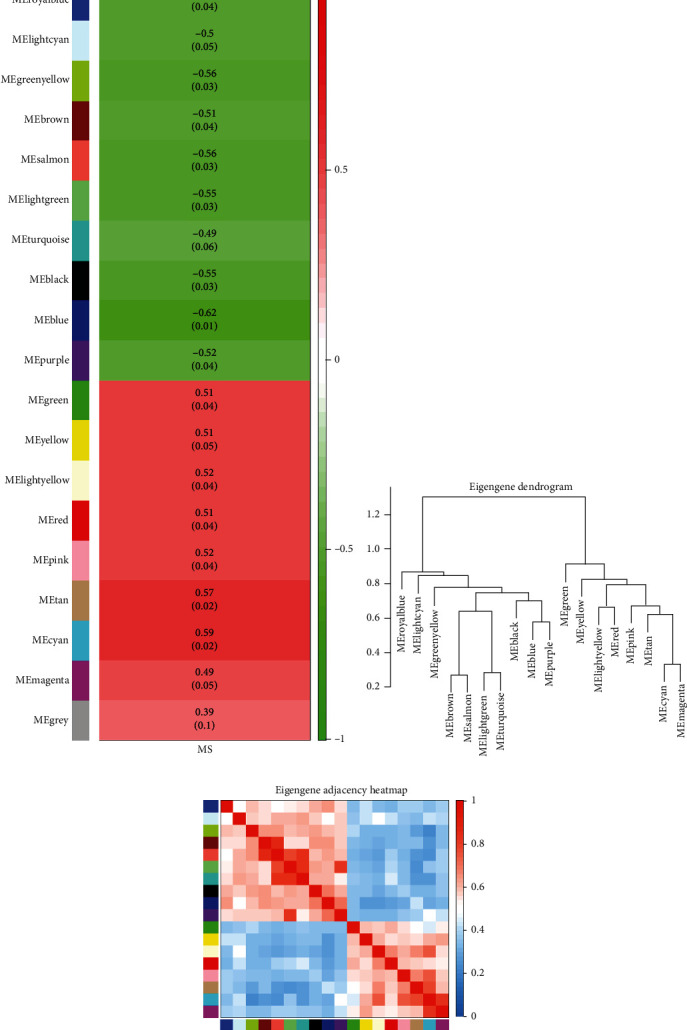
Module-trait relationships and eigengene dendrogram. (a) Module-trait relationships of the genes were calculated based on the correlations between the module eigengenes (MEs) and MS occurrence. The numbers in the rectangle represent the corresponding *P* value and the correlation coefficient. The correlation coefficient of the cyan module is the highest with significance, suggesting that this module is the key module highly related to MS. (b) Correlated hierarchical clustering of the adjacency modules. The branches of the dendrogram group are correlated. (c) Correlated heat map of eigengene adjacency. The light-blue color represents low adjacency, while the red color represents high adjacency. The cyan module exhibits coexpression relationships with the other modules, especially the magenta and tan modules.

A total of 8,819 genes (top 50%) in the GSE52139 dataset were selected to construct coexpression networks using WGCNA after eliminating the genes with insufficient information. The hierarchical clustering dendrogram showed that all 16 samples clustered well without outliers and were divided into two groups. The samples from healthy donors primarily belonged to one cluster (Figure 2(a)). A power of *β* = 8 was selected as the soft threshold to create a scale-free network, as shown in Figure 2(b).  Figure 2(c) shows that 18 coexpression modules were obtained using the average linkage hierarchical clustering algorithm, including 525 genes in the black module, 871 genes in the blue module, 712 genes in the brown module, 555 genes in the cyan module, 580 genes in the green module, 647 genes in the green-yellow module, 263 genes in the light-cyan module, 217 genes in the light-green module, 178 genes in the light-yellow module, 497 genes in the magenta module, 498 genes in the pink module, 438 genes in the purple module, 550 genes in the red module, 162 genes in the royal-blue module, 289 genes in the salmon module, 306 genes in the tan module, 897 genes in the turquoise module, and 630 genes in the yellow module. The remaining four genes that were not found to belong to a module were assigned to the gray module, which was excluded from subsequent analysis. The module-trait relationships shown in Figure 3(a) depict the correlation between MS occurrence and each module in the GSE52139 dataset. The cyan module was found to have a strong positive correlation with MS (*R*^2^ = 0.59, *P* = 0.02). Therefore, as the key module, the cyan module became the focus for further analysis. Figures 3(b) and 3(c) show the eigengene dendrogram and heat map of the eigengene adjacency of modules, respectively. These diagrams demonstrate that the cyan module exhibited coexpression relationships with the other modules, especially the magenta and tan modules.

### 3.2. Functional Enrichment Analyses of the Genes in the Cyan Module

**Figure 4:**
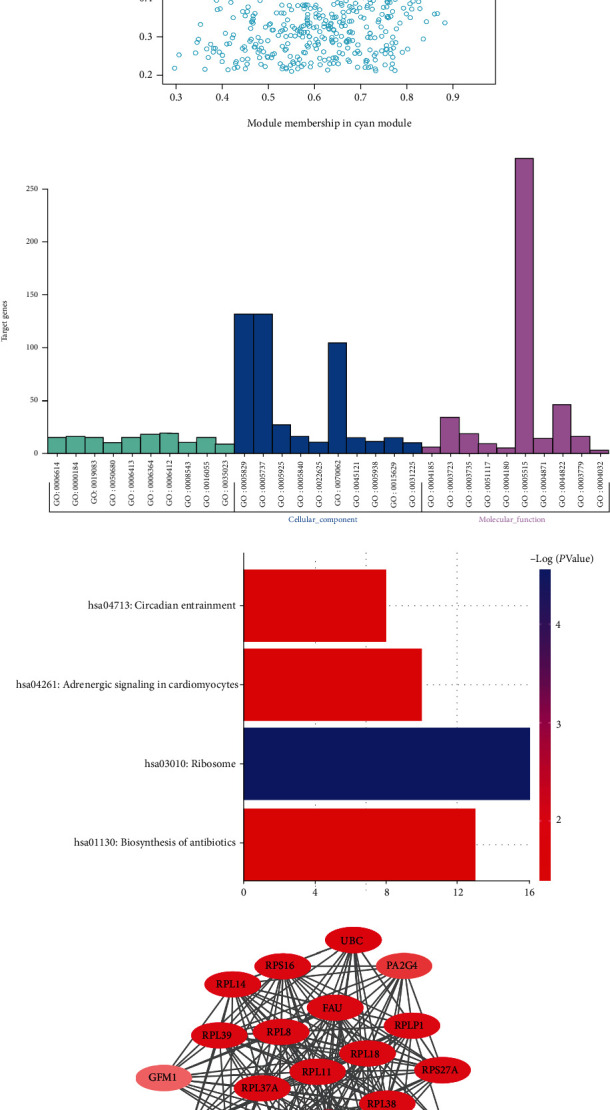
Gene Ontology (GO) enrichment and Kyoto Encyclopedia of Genes and Genomes (KEGG) analyses of the cyan module. (a) Scatterplot of gene significance for MS vs. module membership in the cyan module. The cyan module shows the highest relationship with MS. (b) The top 10 terms of GO categories of genes in the cyan module. The genes in the cyan module are primarily gathered in Biological Process (BP), Cellular Component (CC), and Molecular Function (MF), including “signal recognition particle- (SRP-) dependent cotranslational protein targeting to membrane,” “cytosol,” “serine-type carboxypeptidase activity,” and “positive regulation of GTPase activity.” (c) The terms of KEGG analysis of genes in the cyan module. The enriched KEGG pathways of the cyan module genes include “ribosome,” “circadian entrainment,” and “adrenergic signaling in cardiomyocytes.” (d) Diagram of the protein-protein interaction (PPI) cluster with the highest score based on MCODE. The ellipse represents the gene node, and the connecting line represents the interaction. The color of the ellipse corresponds to its node degree, indicating that the darker the ellipse, the higher the node degree. This protein-protein cluster is composed of 22 nodes and 199 interactive edges. The ribosome-related proteins, such as ribosomal protein L8 (RPL8), RPL11, and RPL14, become the components of the network. *P* < 0.05 indicates significance.

The scatter plots in Figure 4(a) demonstrate the correlation between MM and GS for the clinical traits of the cyan module genes. GO and KEGG analyses were subsequently conducted to gain more knowledge about the gene group function in the key modules using DAVID.  Figure 4(b) shows that the genes in the cyan module were primarily gathered in Biological Process (BP), Cellular Component (CC), and Molecular Function (MF), including “signal recognition particle- (SRP-) dependent cotranslational protein targeting to membrane” (GO:0006614, *P* = 4.22*E* − 07), “cytosol” (GO:0005829, *P* = 6.69*E* − 06), “serine-type carboxypeptidase activity” (GO:0004185, *P* = 2.75*E* − 05), and “positive regulation of GTPase activity” (GO:0043547, *P* = 0.006410677).  Figure 4(c) presents the enriched KEGG pathways of the cyan module genes, which include “ribosome” (hsa03010, *P* = 2.74*E* − 05), “circadian entrainment” (hsa04713, *P* = 0.03262712), and “adrenergic signaling in cardiomyocytes” (hsa04261, *P* = 0.033202664). The results of the GO and KEGG analyses of the cyan module are listed in Supplementary Tables 1 and 2. A PPI network was also established, and a topological assessment was implemented.  Figure 4(d) indicates the protein-protein cluster with the highest score (18.952) based on MCODE in the cyan module, which is composed of 22 nodes and 199 interactive edges. The genes with MCODE scores > 0.4 are listed in Supplementary Table 3.

### 3.3. ceRNA Construction in the Cyan Module

**Figure 5:**
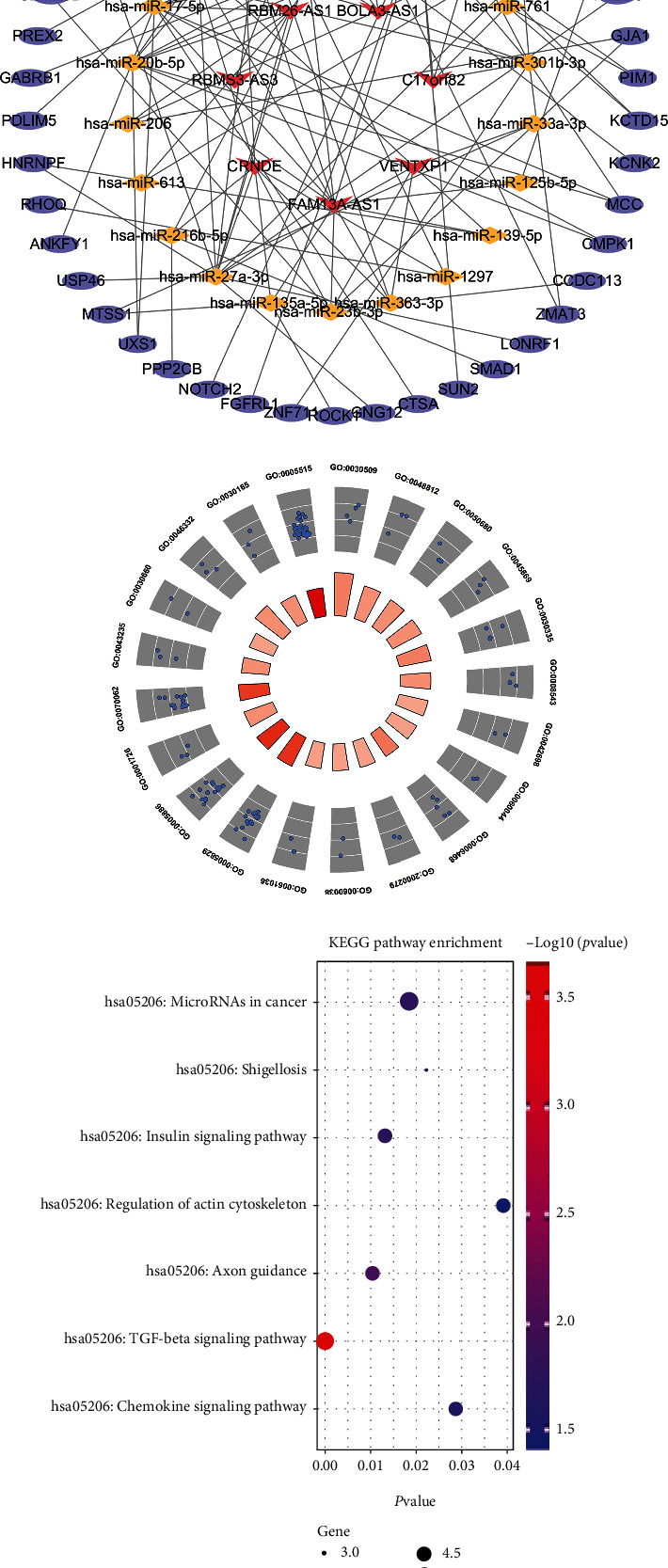
lncRNA-associated competing endogenous RNA (ceRNA) network in the cyan module. (a) The constructed ceRNA network. The red V shape represents lncRNA, the yellow diamond represents miRNA, and the purple ellipse represents mRNA. This ceRNA network is composed of seven lncRNAs, 45 mRNAs, 21 miRNAs, and 111 edges. (b) GO categories for mRNA targets in the ceRNA network. Blue circles represent the genes. The color of the trapezium corresponds to the number of genes in each functional GO term, indicating that the more gathered genes, the darker the trapezium. The circle plot shows that the targeted mRNAs are bound to the “Bone Morphogenetic Proteins (BMPs) signaling pathway,” “cytosol,” and “small mother against decapentaplegic (SMAD) binding.” (c) KEGG terms of the mRNA targets in the ceRNA network. The results reveal that the targeted mRNAs are primarily enriched in the “Transforming Growth Factor-*β* (TGF-*β*) signaling pathway” and “axon guidance.” *P* < 0.05 indicates significance.

The cyan module includes 20 lncRNAs; the potential miRNA and mRNA targets were predicted to determine the regulatory function of these lncRNAs. After cross-matching with 535 mRNAs in the cyan module, 45 mRNAs were identified as target candidates and a network was created, as shown in Figure 5(a). This ceRNA network was composed of seven lncRNAs, 45 mRNAs, 21 miRNAs, and 111 edges, as listed in Table 1. To gain further insight into the targeted mRNAs in the ceRNA network, GO and KEGG analyses were carried out. The complete list of GO analysis terms is presented in Supplementary Table 4. The circle plot in Figure 5(b) shows that the targeted mRNAs were bound to the “Bone Morphogenetic Proteins (BMPs) signaling pathway” (GO:0030509, *P* = 9.65*E* − 04), “cytosol” (GO:0005829, *P* = 0.010823781), and “small mother against decapentaplegic (SMAD) binding” (GO:0046332, *P* = 0.00560239), among others. Interestingly, the KEGG results shown in Figure 5(c) and Supplementary Table 5 reveal that the targeted mRNAs were primarily enriched in the “Transforming Growth Factor-*β* (TGF-*β*) signaling pathway” (hsa04350, *P* = 2.15*E* − 04) and “axon guidance” (hsa04360, *P* = 0.010506171). The node degree of each gene in this ceRNA network was also assessed by employing the built-in “Cytohubba” tool of Cytoscape. The degree of centrality was calculated as a crucial topological factor, reflecting the possibility of one gene being a hub gene [17]. As listed in Table 2, a total of nine genes with node degrees larger than five were discovered, including four lncRNAs (*FAM13A-AS1*, *CRNDE*, *RBM26-AS1*, and *VENTXP1*).

### 3.4. Identification of Real Hub Genes

**Figure 6:**
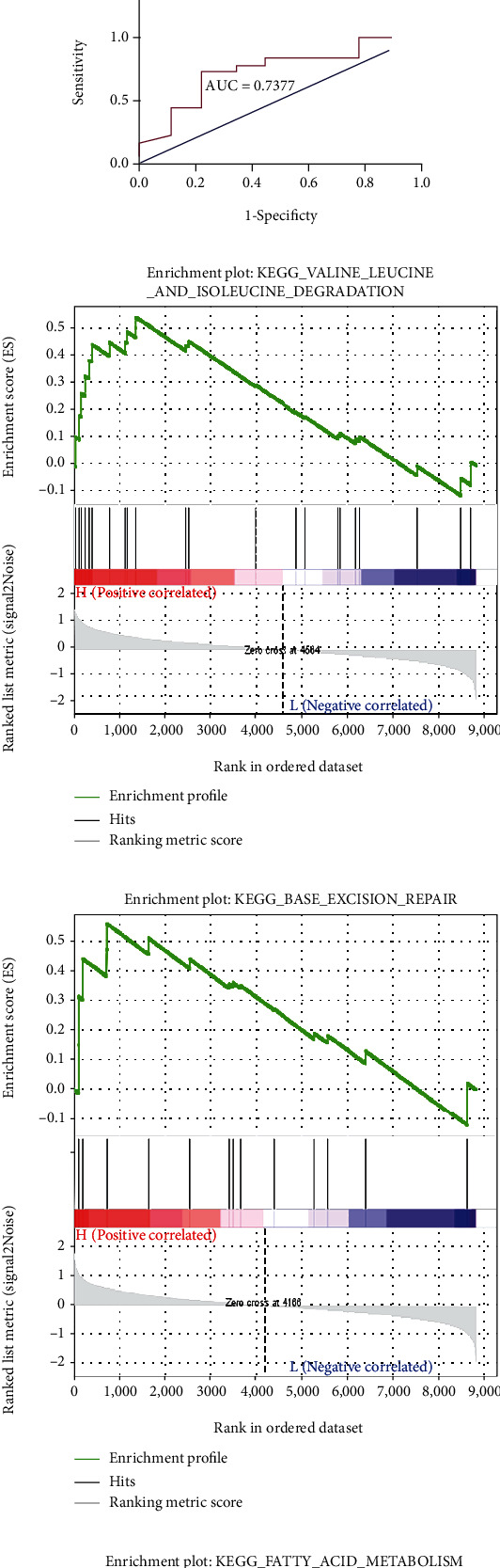
Hub gene validation. (a) Venn diagram used to select the hub candidates. The key genes in the cyan module, hub nodes in the PPI of the cyan module, and the genes in the designed ceRNA network were intersected to obtain 15 common genes. (b) Sub-ceRNA network of hub genes. This network is composed of three hub genes, four lncRNAs, four miRNAs, and 13 interactions. (c) Receiver operating characteristic (ROC) curve of *CYBRD1*. Area under the curve (AUC) = 0.7963, *P* = 0.01359. (d) ROC curve of *GNG12*. AUC = 0.7840, *P* = 0.01802. (e) ROC curve of *SMAD1*. AUC = 0.7377, *P* = 0.04773. (f) The representative result of genomes and gene set enrichment analysis (GSEA) for *CYBRD1*. “Valine leucine and isoleucine degradation” is highly related to a high expression of *CYBRD1*. (g) The representative result of GSEA for *GNG12*. “Base excision repair” is highly related to a high expression of *GNG12*. (h) The representative result of GSEA for *SMAD1*. “Fatty acid metabolism” is highly related to a high expression of *SMAD1*. *P* < 0.05 indicates significance.

Another goal of the current study was to identify hub genes that could be potential biomarkers for MS diagnosis or prognosis. First, because higher MM and GS typically indicate a closer association of the gene with the clinical feature, we filtered the genes in the cyan module based on the following conditions: MM ≥ median (MMcyan ≥ 0.599115) and GS ≥ median (GSMS ≥ 0.29545), resulting in 219 filtered genes. Subsequently, 329 genes from the cyan module, whose MCODE scores were higher than 0.4, were screened out. After cross-matching with the lncRNAs and mRNA targets in the ceRNA network, 15 genes in the cyan module were selected as potential hub genes, as shown in Figure 6(a). ROC curves and AUCs are useful tools for evaluating test accuracy and identifying biomarkers to distinguish between patients with and without a health outcome of interest [18, 19]. Therefore, to acquire true hub genes for MS, ROC curves were generated after testing the expression level of all candidates, and the AUC (95% CIs) was calculated by capitalizing the independent expression profiles in the GSE126802 dataset (Table 3 and Figures 6(c)–6(e)). Based on our criteria, we eventually identified three genes with an elevated expression and high predictive accuracy for MS, namely, *small mother against decapentaplegic 1* (*SMAD1*), *cytochrome b reductase 1* (*CYBRD1*), and *G protein subunit gamma 12* (*GNG12*). Single-gene GSEA revealed the possible molecular cascades triggered by upregulating the hub genes. As shown in Figures 6(f)–6(h), “valine leucine and isoleucine degradation,” “base excision repair,” and “fatty acid metabolism” were highly associated with a high expression of *CYBRD1*, *GNG12*, and *SMAD1*, respectively. Finally, we extracted 13 interactions consisting of four lncRNAs and four miRNAs to generate a hub gene-related ceRNA network, as shown in Figure 6(b).

## 4. Discussion

In this study, we performed WGCNA to systematically evaluate the gene coexpression patterns based on the RNA-sequencing dataset using the spinal cord of patients with MS. We discovered 18 modules, among which the cyan module was markedly aligned with MS. Considering that lncRNAs are involved in MS development, as observed in recent studies, we further constructed an lncRNA-associated ceRNA network in the key module only. This analytical process has been proved to be effective in identifying pivotal lncRNAs, and the ceRNA network constructed is beneficial for exploring the regulatory mechanism in multiple disorders, such as hepatocellular carcinoma and knee osteoarthritis [20, 21]. We also identified hub genes and evaluated their usefulness for the prognosis of MS. Compared with previous reports [22, 23], our study set different criteria for dataset selection and employed new bioinformatics approaches. Briefly, (1) we concentrated on the spinal cord, which is extensively involved in MS but has not been thoroughly investigated in neuropathological studies, and (2) we sought to explore the underlying mechanism by creating a ceRNA network in the key module identified to be significantly correlated with MS. To our knowledge, this is the first report of lncRNA-related ceRNA networks based on WGCNA using spinal cord datasets.

As a chronic AD of the central nervous system, MS affects patients between 20 and 40 years of age, contributing to irreversible neurological disability in young adults [24]. Although the etiology of MS has not been clearly revealed, environmental elements and several susceptible genes are both considered integral to disease pathogenesis. According to our WGCNA results, MS was closely associated with the cyan module. Notably, the genes in this module chiefly gathered to the ribosome-related functions, such as the GO terms “SRP-dependent cotranslational protein targeting to membrane” and “positive regulation of GTPase activity,” as well as the KEGG term “ribosomes.” Ribosomes are the cellular factories responsible for protein production. This process must be under fine control to ensure that the correct protein appears in the proper site, especially in neurons, owing to their unique structure. Accordingly, the implication of ribosome dysfunction in neurological disorders is not surprising. The ribosomal SRP-dependent pathway is essential for guiding proteins to the membrane and for their subsequent insertion into the membrane or release, whereas guanosine triphosphate (GTP) is necessary for this process [25, 26]. In addition to our findings, a number of molecules and pathways involved in ribosome biogenesis have been connected with MS as well, including RNA polymerase I (POL1), nuclear factor kappa-B (NF-*κ*B), and p53 [27, 28]. Acting as an inhibitor of POL1, a new compound, RAM-589.555, was observed to mitigate MS-like syndromes by blocking the ribosomal RNA transcription [29]. However, the immune-related or inflammation-related pathways were not highlighted in our functional assessments, and this is not consistent with previous bioinformatics analyses [22, 30]. The use of different tissue samples for the original data might explain these outcomes. Nonetheless, given the cooccurrence of pro- and anti-inflammatory factors in periplaque demyelinated lesions of the spinal cord, inflammation in the spinal cord might be a relatively chronic process [31]. Thus, based on our findings, we speculate that inflammation in the spinal cord may be initiated through other cellular cascades, rather than being directly activated, in MS.

The ceRNA hypothesis was proposed to explain how RNAs communicate with each other, establishing an interactive network to modify the functional genetic information in physiological and pathological conditions. Briefly, lncRNA and mRNA could competitively combine with mutual miRNA by recognizing miRNA response elements (MREs), constituting lncRNA-miRNA-mRNA connections. Once the expression of lncRNA is elevated, it undergoes “miRNA sponging” to adhere to multiple miRNAs, resulting in fewer miRNAs remaining for coupling with common MREs. Hence, the expression of the corresponding mRNAs is regulated [32, 33]. In terms of MS, the lncRNA, *GM15575*, was demonstrated to be involved in the progression of Th17 cell-associated MS by acting as a ceRNA to suppress *miR-686* activity [34]. Sulfasalazine, an effective medicine for MS, could suppress microglia switching to a proinflammatory phenotype via the ceRNA effect of *miR-136-5p* and the lncRNA *HOTAIR* [35]. Nevertheless, this process is more complicated in reality because one lncRNA typically has numerous miRNA targets and one miRNA has numerous mRNA targets, thereby forming a ceRNA network. A recent study integrated multiple microarray datasets to identify lncRNA-mediated competing triplets in patients with MS [36]. Three lncRNAs, namely, *XIST*, *OIP5-AS1*, and *CTB-89H12.4*, were found to be involved in the pathogenesis but were not detected in our study. This discrepancy may be attributed to different specimen selections. Additionally, we established our ceRNA network based on the key module from WGCNA, instead of on differentially expressed genes, which included the clinic-related genes closely linked with the disorder. Similarly, the distinction of analytical approaches may explain why several lncRNAs investigated in MS research, such as *BDNF-AS*, *GAS5*, *MALAT1*, and *NEAT1* [37–40], did not appear in our constructed ceRNA network. In the current study, 21 miRNAs were predicted using seven lncRNAs in the designed ceRNA network. The level of the gene with the highest node degree, *miR-17-5p*, has been demonstrated to be increased in CD4 (+) cells from patients with MS, implying the reliability of the current analysis [41]. Moreover, circulating *miR-338-3p* has been nominated as a potential MS diagnostic biomarker for patients with radiologically isolated syndrome [42]. Despite the lack of evidence concerning the role of lncRNAs in MS, these noncoding RNAs have been implicated in numerous disease contexts. For instance, several *in silico analyses* have shown that *FAM13A-AS1* is involved in various cancers [43–45]. The knockout of *CRNDE* can alleviate hypoxic-ischemic brain damage by promoting autophagy [46]. *VENTXP1* inhibits tumor growth by suppressing NF-*κ*B signaling in head and neck squamous cell carcinoma [47]. Therefore, the potential function of these lncRNAs in the scenario of MS might not be neglected. On the other hand, compared with the entire cyan module genes, the putative mRNA targets in the ceRNA network were clustered in different functional categories, which were proverbial in MS, such as “BMP signaling pathway,” “SMAD binding,” “TGF-*β* signaling pathway,” and “axon guidance.” BMP and TGF-*β* are both crucial cytokines of the immune system with similar receptors and messengers. Since BMPs primarily play a proinflammatory role and TGF-*β* inhibits inflammation, patients with MS frequently manifest BMP overactivity and the suppression of TGF-*β* signaling [48]. An abnormal expression of SMAD, the exclusive protein responsible for delivering TGF-*β* signals into the nucleus, has been associated with MS progression [49]. Hence, our findings suggest that lncRNAs might crosstalk with their ceRNAs and affect the pathways mentioned above, thereby participating in MS progression.

Another goal of the present study was to identify hub genes as biomarkers with potential diagnostic value and determine their possible connection with MS pathogenesis. Three genes were discovered based on our selected conditions, including a well-documented component of TGF-*β* signaling, the *SMAD1*. Particularly, other molecules were thought to drive the pathology of MS through regulating the TGF-*β* pathway by targeting *SMAD1*. *Homeobox A5* (*HOXA5*) is reported to encode a protein that binds to SMAD1. The physiological overexpression of HOXA5 in the spinal cords of patients with MS may promote the TGF-*β* pathway and favor the slow progression of TGF-*β*1-mediated gliosis [50]. An aberrant expression of *miR-326* and *miR-26a* was proposed to influence the differentiation of pathogenic Th17 cells by SMAD1 in the MS context [51]. It has been illustrated that inhibiting BMP signaling diminished the expression of astrocytic SMAD1 and induced early oligodendrogenesis-mediated remyelination, implying a therapeutic potential in MS [52]. Meanwhile, an acetylcholinesterase inhibitor, donepezil, could promote myelin-forming oligodendrocyte differentiation by suppressing the activation of SMAD1 [53]. In addition, single-gene GSEA disclosed that this gene might be associated with “fatty acid.” A wealth of data support that the perturbations of the intestinal microbiota and microbial products, such as short-chain fatty acids (SCFA), can lead to MS. Furthermore, although the cause of microbiota-induced SCFA dysregulation remains unclear, SCFA supplementation, as an adjuvant treatment, may reduce the relapse rates and brain atrophy in patients [54–56]. Consequently, further examination of the interplay between SMAD1 and SCFA could present unappreciated insights into the potential therapeutic benefits of SCFAs in MS management.

*GNG12* is distributed throughout glial cells and is highly expressed in reactive astrocytes. An upregulation of GNG12 might promote the activity of protein kinase C (PKC), facilitating GNG12 protein phosphorylation and negatively regulating the inflammatory response. By employing bioinformatics methods, GNG12 has been elucidated to play important roles in tumorigenesis, thereby becoming a new prognosis-related biomarker and immunotherapy target [57–59]. Unfortunately, so far, the research with respect to the function of GNG12 in MS is extremely scarce. The only report is that GNG12 could become a novel negative regulator in response to lipopolysaccharide- (LPS-) induced inflammation in the microglial cell line, BV-2, suggesting its potential involvement in MS development [60]. Our GSEA results also revealed that *GNG12* might be associated with “base excision repair,” a highly conserved pathway, which is involved in the maintenance of genomic integrity by correcting the endogenous DNA damage and removing the resulting lesions. Since DNA damage is typically accompanied by immune system activation, “base excision repair” has also been previously reported in AD and neurodegenerative diseases [61, 62]. Therefore, it is reasonable to assume that the pharmacological manipulation of *GNG12* would offer an opportunity for therapeutic intervention in inflammatory disorders, including MS.

Finally, *CYBRD1* is a member of the cytochrome B family that encodes an iron-regulated protein. Similar to *GNG12*, most of the existing researches focusing on this gene are related to cancers [63, 64], and the knowledge regarding the role of *CYBRD1* in MS remains unclear. However, neuropathological data have revealed a skewed distribution of iron in the brains of patients with MS, indicating that the release of iron within active lesions may aggravate demyelination and neurodegeneration [65]. Moreover, the latest research discovered that white matter lesions surrounded by a rim of iron-containing microglia were associated with the severity of disease in patients with MS, highlighting the role of iron metabolism in MS progression [66]. As a regulator of iron homeostasis, it is definitely worthy to explore whether and how CYBRD1 implicates this procedure. Intriguingly, *CYBRD1* was related to “valine leucine and isoleucine degradation” as our GSEA results. These three amino acids belong to the proteinogenic branched-chain amino acids (BCAAs), which have a possible pathophysiological relationship with human iron metabolism and are mediated by the mechanistic target of the rapamycin complex 1 (mTORC1) pathway [67]. Accordingly, more attention should be paid to *CYBRD1* to shed light on the molecular mechanism of MS in future research.

The present study had several limitations that should be discussed. For instance, the sample size of the GSE52139 dataset is relatively small, and owing to the rarity of expression datasets of spinal cord samples, the validation dataset was obtained from human brain samples, which might exclude certain hub genes. Moreover, the conclusions are primarily based on bioinformatics analysis, thereby warranting further experimental confirmation. Laboratory analyses focusing on novel hub genes and molecular pathways using various models or clinical specimens are ultimately needed.

## 5. Conclusion

In conclusion, our study focused on the crucial gene group that is closely associated with the MS clinical features generated using WGCNA to explore the MS mechanism occurring in the spinal cord. The lncRNA-associated ceRNA network constructed in the key module revealed the regulatory role of lncRNAs. To our knowledge, this is the first study to design a ceRNA network related to MS based on the WGCNA algorithm using a spinal cord dataset. The predicted mRNA targets of lncRNAs have distinct biological functions compared with other genes in the key module, implying the involvement of multiple pathophysiological pathways. Moreover, hub genes with diagnostic values, including *SMAD1*, *CYBRD1*, and *GNG12*, were identified. Our findings contribute to a comprehensive understanding of MS development in the spinal cord, particularly from the perspective of lncRNAs. Additionally, the hub genes identified herein might not only be novel biomarkers for the diagnosis and prognosis of MS but also be possible therapeutic targets for disease management.

## Figures and Tables

**Table 1:** The information of lncRNA-associated ceRNA network in cyan module.

lncRNAs (7)	C17orf82, BOLA3-AS1, RBM26-AS1, RBMS3-AS3, CRNDE, FAM13A-AS1, VENTXP1

miRNAs (21)	hsa-miR-301b-3p, hsa-miR-363-3p, hsa-miR-17-5p, hsa-miR-20b-5p, hsa-miR-24-3p, hsa-miR-761, hsa-miR-3619-5p, hsa-miR-206, hsa-miR-27a-3p, hsa-miR-125b-5p, hsa-miR-107, hsa-miR-1297, hsa-miR-129-5p, hsa-miR-429, hsa-miR-23b-3p, hsa-miR-338-3p, hsa-miR-33a-3p, hsa-miR-216b-5p, hsa-miR-139-5p, hsa-miR-613, hsa-miR-135a-5p

mRNA targets (45)	ZMAT3, CCDC113, CMPK1, MCC, KCNK2, KCTD15, PIM1, GJA1, TMED5, BMPR1B, WEE1, CYBRD1, MAPK1, CRKL, PHKA1, DICER1, SEMA7A, RRAGD, ABHD17C, TOB1, FOXP4, ADD3, CMTM4, TGFBR3, ARHGEF26, SEMA6D, PREX2, GABRB1, PDLIM5, HNRNPF, RHOQ, ANKFY1, USP46, MTSS1, UXS1, PPP2CB, NOTCH2, FGFRL1, ZNF711, ROCK1, GNG12, CTSA, SUN2, SMAD1, LONRF1

**Table 2:** The genes with node degree higher than 5 in ceRNA network.

Gene name	Gene type	Node degree
hsa-miR-17-5p	miRNA	13
hsa-miR-27a-3p	miRNA	13
FAM13A-AS1	lncRNA	11
hsa-miR-301b-3p	miRNA	9
hsa-miR-107	miRNA	9
hsa-miR-20b-5p	miRNA	9
CRNDE	lncRNA	8
RBM26-AS1	lncRNA	7
VENTXP1	lncRNA	6

**Table 3:** The validation of the potential hub genes of MS.

Gene	AUC	*P*-Value^#^	95%CI^##^	*P*-Value^###^	Significance
CYBRD1	**0.7963**	**0.01359**	**0.5833 to 1.009**	**0.0103**	∗
GNG12	**0.7840**	**0.01802**	**0.5867 to 0.9812**	**0.0151**	∗
SMAD1	**0.7377**	**0.04773**	**0.5336 to 0.9418**	**0.0268**	∗
ROCK1	0.5741	0.5371	0.3370 to 0.8111	0.4465	n.s
ADD3	0.7099	0.08039	0.4907 to 0.9291	0.0937	n.s
GABRB1	0.5154	0.8977	0.2364 to 0.7945	0.9662	n.s
TGFBR3	0.6265	0.2917	0.3689 to 0.8842	0.3381	n.s
GJA1	0.6759	0.1427	0.4275 to 0.9244	0.1850	n.s
PHKA1	0.6975	0.09985	0.4661 to 0.9290	0.4950	n.s
RRAGD	0.6883	0.1168	0.4815 to 0.8950	0.1138	n.s
BMPR1B	0.6327	0.2688	0.3808 to 0.8847	0.4719	n.s
LONRF1	0.5062	0.9590	0.2720 to 0.7404	0.7454	n.s
SEMA6D	0.5494	0.6807	0.3125 to 0.7862	0.6113	n.s
CMTM4	0.5216	0.8571	0.2853 to 0.7579	0.8389	n.s
TOB1	0.5000	1.0000	0.1183 to 0.8817	0.7298	n.s

# *P*-value of AUC. ## 95% confidence interval of AUC. ### *P*-value of gene expression comparison calculated by un-paired t-test (two-tailed), normal vs patients. ∗*P*<0.05. n.s no significance.

## Data Availability

The data used to support the findings of this study are included within the article.

## Supplementary Materials

Supplementary 1 Supplementary Table 1: GO analysis of genes in cyan module.

Supplementary 2 Supplementary Table 2: KEGG analysis of genes in cyan module.

Supplementary 3 Supplementary Table 3: the genes with MCODE score higher than 0.4.

Supplementary 4 Supplementary Table 4: GO analysis of mRNA targets in ceRNA network.

Supplementary 5 Supplementary Table 5: KEGG analysis of mRNA targets in ceRNA network.
