# Supplementary material for: Identification of Novel Key Genes and Pathways in Multiple Sclerosis Based on Weighted Gene Coexpression Network Analysis and Long Noncoding RNA-Associated Competing Endogenous RNA Network
Source: Oxid Med Cell Longev. 2022 Mar 2;2022:9328160. doi: 10.1155/2022/9328160 (PMC8915924; doi:10.1155/2022/9328160)
Supplement: Supplementary 1 — Supplementary Table 1: GO analysis of genes in cyan module. [file 9328160.f1.docx]

**Supplementary Table1. GO analysis of genes in cyan module**

| **Term** | **Count** | **PValue** | **Genes** |
| --- | --- | --- | --- |
| GO:0006614~SRP-dependent cotranslational protein targeting to membrane | 15 | 4.22E-07 | RPLP1, RPS6, RPL11, RPL10A, RPL8, RPS16, RPS29, RPL37A, RPL14, RPL38, FAU, RPS27A, RPL18, UBA52, RPL39 |
| GO:0000184~nuclear-transcribed mRNA catabolic process, nonsense-mediated decay | 16 | 1.48E-06 | RPLP1, RPS6, RPL11, RPL10A, RPL8, SMG5, RPS16, RPS29, RPL37A, RPL14, RPL38, FAU, RPS27A, RPL18, UBA52, RPL39 |
| GO:0019083~viral transcription | 15 | 3.65E-06 | RPLP1, RPS6, RPL11, RPL10A, RPL8, RPS16, RPS29, RPL37A, RPL14, RPL38, FAU, RPS27A, RPL18, UBA52, RPL39 |
| GO:0050680~negative regulation of epithelial cell proliferation | 10 | 2.67E-05 | SOX2, TGFBR3, AR, RGN, PEX2, SOX9, MCC, CDC73, MTSS1, EREG |
| GO:0006413~translational initiation | 15 | 3.75E-05 | RPLP1, RPS6, RPL11, RPL10A, RPL8, RPS16, RPS29, RPL37A, RPL14, RPL38, FAU, RPS27A, RPL18, UBA52, RPL39 |
| GO:0006364~rRNA processing | 18 | 1.43E-04 | RPLP1, RPS6, RPL11, PA2G4, RPL10A, RPL8, RPS16, RPS29, RPL37A, RPL14, RPL38, FAU, RPS27A, RPL18, RPP14, UBA52, RPL39, DCAF13 |
| GO:0006412~translation | 19 | 3.62E-04 | RPLP1, MRPL19, RPS6, RPL11, RPL10A, RPL8, MRPL43, SLC25A18, RPS16, SLC25A39, RPS29, RPL37A, RPL14, RPL38, FAU, RPS27A, RPL18, UBA52, RPL39 |
| GO:0008543~fibroblast growth factor receptor signaling pathway | 10 | 5.37E-04 | GALNT3, FLRT3, KIF16B, HNRNPF, UBC, MAPK1, FGF1, RPS27A, UBA52, FGFRL1 |
| GO:0016055~Wnt signaling pathway | 15 | 9.71E-04 | CTNND2, ETV2, ZBTB33, MCC, NDRG2, CCAR2, CDC73, DKK3, PYGO1, TMEM88, UBC, CPE, CD24, RPS27A, UBA52 |
| GO:0035023~regulation of Rho protein signal transduction | 9 | 0.002149825 | PREX2, PLEKHG3, FARP1, ARHGEF9, FGD6, ARHGEF26, ARHGDIA, ARHGEF1, MYO9B |
| GO:0006518~peptide metabolic process | 4 | 0.005329386 | CPXM2, CPXM1, AEBP1, PAM |
| GO:0005978~glycogen biosynthetic process | 5 | 0.0059392 | PPP1R3C, UBC, RPS27A, GYG2, UBA52 |
| GO:0043547~positive regulation of GTPase activity | 28 | 0.006410677 | SPTBN4, ARHGEF26, AGAP1, ARHGAP17, CSF2RB, FGF1, PREX2, FGD6, RASGEF1A, ARHGDIA, CHN1, RGS20, RGS22, FBXO8, CYTH1, S100A10, PLEKHG3, FARP1, RAB3IP, MYO9B, ADAP1, EREG, ARHGAP32, ARHGEF9, CDC42EP4, RGN, ARHGEF1, PLCB1 |
| GO:0090002~establishment of protein localization to plasma membrane | 6 | 0.00659482 | SPTBN4, TTC8, ROCK1, RDX, EZR, S100A10 |
| GO:0090090~negative regulation of canonical Wnt signaling pathway | 12 | 0.007151097 | SOX2, TMEM88, CAV1, HECW1, UBC, PSME4, RGS20, SOX9, MCC, RPS27A, UBA52, DKK3 |
| GO:0030203~glycosaminoglycan metabolic process | 5 | 0.008822933 | SDC4, B3GAT2, SDC2, GPC4, GPC6 |
| GO:0061036~positive regulation of cartilage development | 4 | 0.009793625 | SMAD1, SOX9, SOX6, BMPR1B |
| GO:0032781~positive regulation of ATPase activity | 5 | 0.01117825 | TPM1, RGN, DNAJC9, ATP1B1, PFN2 |
| GO:0003091~renal water homeostasis | 5 | 0.012493642 | WFS1, AQP4, ADCY2, ADCY8, AQP1 |
| GO:0051260~protein homooligomerization | 12 | 0.01281179 | LGI1, KCNF1, KCNG3, CAV1, AKR1C1, DPYSL3, EIF2AK3, FAS, MICU1, PAM, KCTD15, FGFRL1 |
| GO:0008284~positive regulation of cell proliferation | 23 | 0.014854523 | YAP1, NTRK2, AKR1C3, TNC, MST1R, PROX1, FGF1, PBX1, WDR77, EREG, CRKL, AR, UFL1, EDNRB, TNFSF4, ID4, MAPK1, BIRC6, CD47, SOX9, CNOT8, MTA3, CACUL1 |
| GO:0060740~prostate gland epithelium morphogenesis | 3 | 0.015505341 | AR, ID4, TNC |
| GO:0032753~positive regulation of interleukin-4 production | 4 | 0.01832805 | IL33, TNFSF4, LGALS9, HAVCR2 |
| GO:0044597~daunorubicin metabolic process | 3 | 0.020286904 | AKR7A2, AKR1C1, AKR1C3 |
| GO:0044598~doxorubicin metabolic process | 3 | 0.020286904 | AKR7A2, AKR1C1, AKR1C3 |
| GO:0060371~regulation of atrial cardiac muscle cell membrane depolarization | 3 | 0.020286904 | GJA1, SCN1B, CACNA1G |
| GO:0048813~dendrite morphogenesis | 5 | 0.020534593 | PREX2, FARP1, SDC2, SLITRK5, DCLK1 |
| GO:0051894~positive regulation of focal adhesion assembly | 4 | 0.020939322 | COL16A1, SDC4, ROCK1, S100A10 |
| GO:0000717~nucleotide-excision repair, DNA duplex unwinding | 4 | 0.023743551 | CETN2, UBC, RPS27A, UBA52 |
| GO:0042448~progesterone metabolic process | 3 | 0.025596244 | AKR1C1, AKR1C3, AFP |
| GO:0000715~nucleotide-excision repair, DNA damage recognition | 4 | 0.026740647 | CETN2, UBC, RPS27A, UBA52 |
| GO:0001649~osteoblast differentiation | 8 | 0.029295754 | SOX2, UFL1, SEMA7A, GJA1, MYBBP1A, CAT, TNC, ALPL |
| GO:0007416~synapse assembly | 6 | 0.029786304 | FARP1, GPM6A, FLRT3, PCDHB6, RAB29, NRXN3 |
| GO:0001523~retinoid metabolic process | 6 | 0.029786304 | SDC4, AKR1C1, SDC2, AKR1C3, GPC4, GPC6 |
| GO:0006297~nucleotide-excision repair, DNA gap filling | 4 | 0.029930004 | POLD2, UBC, RPS27A, UBA52 |
| GO:0000902~cell morphogenesis | 6 | 0.031668387 | YAP1, TENM3, SHROOM1, SOX6, FRY, CLU |
| GO:0060135~maternal process involved in female pregnancy | 4 | 0.033310534 | CNR1, LGALS9, PAM, HAVCR2 |
| GO:0030512~negative regulation of transforming growth factor beta receptor signaling pathway | 6 | 0.035651537 | TGFBR3, CAV1, CD109, UBC, RPS27A, UBA52 |
| GO:0006974~cellular response to DNA damage stimulus | 12 | 0.036581033 | CTC1, YAP1, SHPRH, USP16, NEK4, ZMAT3, BRAT1, MAPKAPK2, PSME4, MAPK1, FMN2, CCAR2 |
| GO:0007605~sensory perception of sound | 9 | 0.036848315 | TUB, SPTBN4, TMIE, WFS1, HOMER2, LRIG1, RPL38, NAV2, ALDH7A1 |
| GO:0006027~glycosaminoglycan catabolic process | 4 | 0.036880708 | SDC4, SDC2, GPC4, GPC6 |
| GO:0007016~cytoskeletal anchoring at plasma membrane | 3 | 0.037664263 | SPTBN4, TLN2, EZR |
| GO:0061045~negative regulation of wound healing | 3 | 0.037664263 | GJA1, CD109, CASK |
| GO:0030857~negative regulation of epithelial cell differentiation | 3 | 0.037664263 | YAP1, CAV1, SOX9 |
| GO:0010628~positive regulation of gene expression | 14 | 0.037812316 | NTRK2, SMAD1, CAV1, RDX, EIF2AK3, TNC, ALOX12B, ANK2, POU3F1, AR, GJA1, CNTN1, LGALS9, EZR |
| GO:0000165~MAPK cascade | 14 | 0.037812316 | SPTBN4, SMAD1, CAV1, CSF2RB, FGF1, EREG, RASGEF1A, DOK5, MAPKAPK2, UBC, PSME4, MAPK1, RPS27A, UBA52 |
| GO:0034220~ion transmembrane transport | 12 | 0.038868819 | ANO1, GABRB1, GJA1, ARHGEF9, GJD3, UBC, AQP4, ANO6, ATP1B1, RPS27A, UBA52, RYR3 |
| GO:0010976~positive regulation of neuron projection development | 7 | 0.041756824 | NTRK2, TENM3, CNR1, DPYSL3, CNTN1, PRKD1, SCN1B |
| GO:0019068~virion assembly | 3 | 0.044359631 | UBC, RPS27A, UBA52 |
| GO:0010939~regulation of necrotic cell death | 3 | 0.044359631 | UBC, RPS27A, UBA52 |
| GO:2000279~negative regulation of DNA biosynthetic process | 3 | 0.044359631 | GJA1, RGN, KCNK2 |
| GO:1900181~negative regulation of protein localization to nucleus | 3 | 0.044359631 | SIN3A, DCLK2, DCLK1 |
| GO:0071230~cellular response to amino acid stimulus | 5 | 0.044497119 | NTRK2, COL16A1, RRAGD, COL4A6, SH3BP4 |
| GO:0006378~mRNA polyadenylation | 4 | 0.044581871 | NUDT21, AHCYL1, GRSF1, CDC73 |
| GO:0032689~negative regulation of interferon-gamma production | 4 | 0.044581871 | IL33, TNFSF4, LGALS9, HAVCR2 |
| GO:0007219~Notch signaling pathway | 8 | 0.046534849 | APH1A, NOTCH2, UBC, CNTN1, ETV2, SOX9, RPS27A, UBA52 |
| GO:0008360~regulation of cell shape | 9 | 0.047456861 | STRIP2, FGD6, CDC42EP4, ARHGDIA, TPM1, RDX, PARVA, EZR, RHOQ |
| GO:0006294~nucleotide-excision repair, preincision complex assembly | 4 | 0.048707903 | CETN2, UBC, RPS27A, UBA52 |
| GO:0033138~positive regulation of peptidyl-serine phosphorylation | 6 | 0.049387022 | NTRK2, CAV1, CREBL2, PRKD1, CD44, PFN2 |
| GO:0005829~cytosol | 132 | 6.69E-06 | AHCYL1, WWC1, HNMT, RPL10A, FGF1, RPL8, CLU, CRKL, SOX2, HERC5, AKR7A2, GJA1, RPS16, CDC23, TMEM88, ARHGDIA, DPYSL3, ANKFY1, RPL38, NBN, GYG2, NUDT15, RPL39, KPNA1, WSB1, TPM1, RPS6, ANGEL1, CASK, ANK2, PHKA1, DICER1, ATG12, WDR77, AR, CACNB4, PPP1R3C, KIF16B, HECW1, MAPKAPK2, PSME4, RRAGD, CMPK1, PRKD1, EZR, UBA52, ALDH7A1, SKAP2, SPTBN4, AK1, GMPR, ARHGAP17, NDRG2, DYNC2LI1, PRDX1, UBC, THEM4, LONRF1, HMGCLL1, GSTM3, SMAD1, RAB3IP, PARVA, LSM5, PAICS, LSM3, AZIN1, ARHGAP32, ARPC2, RPS29, ZFAT, CDC16, OGDH, CYCS, CNOT8, GSTM5, RPLP1, CETN2, FHL1, FMN2, SMG5, UFL1, PLCZ1, RASGEF1A, ACP5, BBS7, MYBPC1, PGAM1, ARHGEF9, DDAH1, EEF1D, RPL37A, CAT, RGN, ARHGEF1, PLCB1, RHOQ, YAP1, PRPS1, AHNAK, ROCK1, RPL11, PBXIP1, ALOX12B, ADD3, CLN5, PPP2CB, CHN1, RPL14, RAB29, MAPK1, CKB, RPS27A, PDLIM5, RPL18, SH3BGR, CYTH1, TUB, NTRK2, FARP1, SORT1, AKR1C1, AKR1C3, BBOX1, MYO9B, TTC8, FABP5, FAS, NCAPD2, FAU, PFKM, TJP2 |
| GO:0005737~cytoplasm | 188 | 1.98E-05 | AHCYL1, SPARC, HHIP, CTNND2, WWC1, AQP4, HNMT, RPL10A, RPL8, CLU, CCAR2, AQP1, SOX2, HERC5, PPP4R4, ARHGDIA, DPYSL3, PIM1, BAALC, DIP2B, LGALS9, TGM5, KPNA1, DNAAF2, LMO3, SEMA6D, RPS6, THOC1, SPICE1, CASK, ZBTB33, ACTN4, DICER1, ATG12, WDR77, DHX40, AR, RAB30, DOK3, EWSR1, HECW1, MAP1A, MAPKAPK2, RRAGD, CMPK1, TLN2, PRKD1, EZR, ALDH7A1, PFN2, SKAP2, SPTBN4, RNASEH2A, AK1, GMPR, AGAP1, ARHGAP17, AEBP1, NDRG2, CACNA1G, STRIP2, DPP8, FLRT3, PRDX1, PCBP1, UBC, KCNN3, NXNL2, CD99, GSTM3, SMAD1, NEK4, HOMER2, RDX, EIF2AK3, DDIT4L, DCLK2, PARVA, ERLIN2, PA2G4, ADAP1, MCC, PAICS, HOPX, AZIN1, PBX1, LRP2BP, PLCXD3, AMACR, ARPC2, RPS29, HNRNPF, CDC16, ID4, TCF3, ALDH18A1, GRSF1, DAZAP2, GABRB1, RPLP1, HSPB8, CCDC124, FHL1, SYNE4, GPHN, SMG5, CDC73, UFL1, SIPA1L1, SIN3A, TMSB4X, EMILIN3, CEP170B, BRD2, DDX59, ACTR5, PGAM1, TFE3, CCDC113, SHROOM1, PROX1, MTSS1, TGFBR3, ANO1, IMPACT, ARHGEF9, MMP14, MDFIC, EEF1D, GAN, CDC42EP4, RGN, ARHGEF1, DNAJC9, PMP2, BIRC7, NAT8L, PLCB1, CD44, KANK2, YAP1, USP16, TOMM40, AHNAK, BRAT1, RPL11, MOSPD1, ADCY2, AFP, ADD3, TOB1, PPP2CB, ABLIM1, FGD6, KCTD20, CHN1, RPL14, CDC37L1, RAB29, LRRC8D, SH3BP4, RGS20, MAPK1, RGS22, CKB, SAMD1, RPS27A, RPP14, PDLIM5, RPL18, MTA3, DCAF13, CYTH1, TUB, FARP1, AKR1C3, SAMD4B, PDE4DIP, MYO9B, HNRNPAB, WEE1, MYBBP1A, FABP5, FAS, NCAPD2, PPIL4, PFKM, TJP2 |
| GO:0005925~focal adhesion | 27 | 3.66E-05 | SDC4, AHNAK, RPLP1, FHL1, TNC, RPL10A, RPL8, GJA1, RPS16, FLRT3, MAPK1, RPL38, RPL18, CD99, CAV1, RDX, CASK, PARVA, ACTN4, MMP14, ARPC2, RPS29, CAT, RPL37A, TLN2, EZR, CD44 |
| GO:0005840~ribosome | 16 | 5.97E-05 | RPLP1, MRPL19, RPS6, RPL11, RPL10A, RPL8, MRPL43, RPS16, RPS29, RPL37A, RPL14, RPL38, FAU, RPS27A, RPL18, UBA52 |
| GO:0022625~cytosolic large ribosomal subunit | 10 | 1.02E-04 | RPLP1, RPL11, RPL37A, RPL14, RPL38, RPL10A, RPL8, RPL18, RPL39, UBA52 |
| GO:0070062~extracellular exosome | 106 | 6.32E-04 | MTCH2, AHCYL1, UXS1, PLOD2, JADE2, HNMT, RPL10A, CLU, ANTXR1, AQP1, CRKL, AKR7A2, GJA1, RPS16, ARHGDIA, ANKFY1, DIP2B, LGALS9, APLP2, ACTN4, ATP1B1, MAPKAPK2, CMPK1, SPARCL1, ZNF711, EZR, UBA52, ALDH7A1, CFB, PFN2, GPM6A, SPTBN4, LRRC57, SDC4, AK1, AEBP1, NDRG2, ZNHIT6, PRDX1, TSPAN6, PCBP1, UBC, TSPAN3, S100A10, CYP2J2, CTSA, GSTM3, NEBL, RDX, CYBRD1, ERLIN2, PA2G4, GNG12, PAICS, GNAL, ARPC2, RPS29, CNTN1, CPE, ALPL, PAM, RPLP1, DBI, ALDH2, SCRN2, ACP5, HAVCR2, PGAM1, NME3, ANO6, TGFBR3, HADHB, ANO1, DDAH1, RPL37A, CAT, VWA1, PMP2, CD47, PLCB1, RHOQ, CD44, TOMM40, AHNAK, RPL11, PRCP, CLN5, PPP2CB, MGAT5, RPL14, RAB29, SH3BP4, MAPK1, CKB, GPC4, RPS27A, CD55, GALNT3, GOLM1, AKR1C1, AKR1C3, BBOX1, SVIP, FABP5, FAS, PFKM |
| GO:0045121~membrane raft | 15 | 0.001833558 | SDC4, AHNAK, CAV1, ADCY2, ANK2, ERLIN2, GJA1, EDNRB, CNR1, CNTN1, FAS, CD24, CD55, RHOQ, S100A10 |
| GO:0005938~cell cortex | 11 | 0.002209644 | ARHGAP32, NDFIP1, CAV1, RGS20, MYO9B, PRKD1, FMN2, FRY, FGF1, ADD3, ASTN2 |
| GO:0015629~actin cytoskeleton | 15 | 0.003082582 | AHNAK, CASK, MYO9B, PARVA, FMN2, ACTN4, MTSS1, ARHGAP32, ABLIM1, ARPC2, CDC42EP4, ZNF74, TLN2, EZR, PDLIM5 |
| GO:0031225~anchored component of membrane | 10 | 0.00414386 | SEMA7A, NRN1, CD109, NTM, CNTN1, ALPL, GPC4, GPC6, SVIP, CD55 |
| GO:0005614~interstitial matrix | 4 | 0.004889676 | CCDC80, TNC, VWA1, NAV2 |
| GO:0015935~small ribosomal subunit | 5 | 0.00612246 | RPS16, RPS29, RPS6, FAU, RPS27A |
| GO:0043209~myelin sheath | 11 | 0.009772698 | PGAM1, PRDX1, RDX, CNTN1, PMP2, NDUFS1, CKB, ATP1B1, PLCB1, EZR, RPS27A |
| GO:0010008~endosome membrane | 12 | 0.014096987 | SUN2, NTRK2, ARHGAP32, NDFIP1, SORT1, UBC, GOLIM4, ANKFY1, VPS45, RPS27A, ANTXR1, UBA52 |
| GO:0005887~integral component of plasma membrane | 54 | 0.014753928 | GABRB1, TENM3, HHIP, AQP4, AQP1, GJA1, EDNRB, SLC16A4, KCNH5, SEMA6D, SLC39A12, EREG, TGFBR3, SLCO1C1, MMP14, GJD3, MEP1B, NRG3, CD47, PRKD1, SLC18B1, CD44, NOTCH2, ADCYAP1R1, STEAP1B, SDC4, NRXN3, CSF2RB, ADCY2, MST1R, LPAR4, APH1A, FLRT3, CNR1, TSPAN6, LRRC8D, GPC4, TSPAN3, GPC6, CD99, CD55, NTRK2, GOLM1, CAV1, GPR1, GPR6, DCLK1, TNFSF4, PCDHB6, FAS, BMPR1B, KCNK2, HCN2, CD200 |
| GO:0005615~extracellular space | 51 | 0.02015139 | SPARC, CPXM2, TNC, FGF1, CLU, LIPC, ARHGDIA, DPYSL3, CPXM1, LGALS9, RPL39, IFNA16, ACTN4, EREG, F5, DKK3, TGFBR3, MEP1B, NRG3, CD109, CAT, SPARCL1, VWA1, DNAJC9, EZR, CRELD2, UBA52, CFB, SEMA7A, NRN1, AEBP1, AFP, FLRT3, PRDX1, UBC, CKB, GPC4, RPS27A, GPC6, LGI1, IL33, CMTM4, GOLM1, RDX, TNFSF4, CPE, ALPL, FAU, CMTM2, PAM, HAMP |
| GO:0030315~T-tubule | 5 | 0.020305508 | AHNAK, RDX, ANK2, EZR, SCN1B |
| GO:0005794~Golgi apparatus | 35 | 0.024595997 | HSPB8, GLIS3, GOLIM4, DBI, NDRG2, CLU, FGFRL1, LMF1, CLN5, APH1A, GJA1, AKR7A2, FGD6, RAB29, MAPK1, MGAT2, SLC39A7, TMED5, CHST6, GALNT3, SORT1, GOLM1, SLC35C1, PDE4DIP, WDR77, MDFIC, ARPC2, CAT, FIBIN, CPE, PRKD1, BIRC7, VPS45, CRELD2, CD44 |
| GO:0005801~cis-Golgi network | 5 | 0.028261784 | RAB30, ANGEL1, GOLIM4, RAB29, TMED5 |
| GO:0009986~cell surface | 24 | 0.028633691 | NOTCH2, SRPX, ADCYAP1R1, SPARC, KCNH5, SDC4, SORT1, SDC2, HHIP, ANO6, MST1R, CLU, ANTXR1, TGFBR3, GJD3, TNFSF4, CD109, FAS, CD24, KCNK2, PAM, CD44, CD55, HAVCR2 |
| GO:0005856~cytoskeleton | 18 | 0.030011981 | FARP1, SLC30A9, ROCK1, TPM1, RDX, RAB3IP, PARVA, ANK2, ADD3, GPHN, FGD6, SIPA1L1, GAN, ARHGDIA, RAB29, MAPK1, EZR, PFN2 |
| GO:0016020~membrane | 76 | 0.031053143 | SRPX, MTCH2, TENM3, TNC, GOLIM4, AQP4, RPL10A, RPL8, SPRED3, LAPTM4B, UFL1, RPS16, CREB3L3, NNT, ANKFY1, BAALC, DIP2B, BBS7, SLC39A7, EMD, SLC16A4, APLP2, PGAM1, RPS6, ANO6, PEX2, ATP1B1, F5, MEP1B, ACOX1, CDH10, CAT, BIRC6, NDUFS1, EZR, YAP1, NOTCH2, SPTBN4, SEMA7A, ADCYAP1R1, LRRC57, AHNAK, BRAT1, RPL11, AGAP1, ADCY2, ADCY8, ADD3, APH1A, PCBP1, RPL14, CDH24, LRRC8D, MGAT2, RPS27A, PDLIM5, RPL18, HMGCLL1, CTSA, GALNT3, CAV1, EIF2AK3, MYO9B, PA2G4, PAICS, CCDC88B, TTC8, MYBBP1A, HNRNPF, CNTN1, FAS, ALPL, NCAPD2, BMPR1B, CD24, PAM |
| GO:0005884~actin filament | 6 | 0.033744266 | TPM1, MYO9B, FMN2, GNG12, EZR, RHOQ |
| GO:0014704~intercalated disc | 5 | 0.035287591 | GJA1, ANK2, ATP1B1, HAMP, SCN1B |
| GO:0001726~ruffle | 7 | 0.03857731 | FGD6, ARHGEF26, ROCK1, RDX, TLN2, EZR, MTSS1 |
| GO:0060170~ciliary membrane | 5 | 0.04048346 | TTC8, HHIP, CASK, BBS7, TMEM231 |
| GO:0022627~cytosolic small ribosomal subunit | 5 | 0.043235552 | RPS16, RPS29, RPS6, FAU, RPS27A |
| GO:0004185~serine-type carboxypeptidase activity | 6 | 2.75E-05 | CTSA, CPXM2, CPE, CPXM1, PRCP, AEBP1 |
| GO:0003723~RNA binding | 34 | 3.16E-05 | AHCYL1, RNASEH2A, RPL11, RPL10A, HTATSF1, RPL8, ELAVL3, RPS16, ZMAT1, ZMAT3, SIN3A, PCBP1, ADAD1, RPL14, RPL38, RPP14, RPL18, RPL39, RBM17, DDX59, THOC1, SAMD4B, TRNT1, LSM5, PUS7, HNRNPAB, LSM3, NUDT21, EWSR1, HNRNPF, ZNF74, FAU, GRSF1, CNOT8 |
| GO:0003735~structural constituent of ribosome | 19 | 5.63E-05 | RPLP1, MRPL19, RPS6, RPL11, RPL10A, RPL8, MRPL43, SLC25A18, RPS16, SLC25A39, RPS29, RPL37A, RPL14, RPL38, FAU, RPS27A, RPL18, UBA52, RPL39 |
| GO:0051117~ATPase binding | 9 | 0.001068185 | AR, WFS1, ZNHIT6, CAV1, RDX, ANK2, ATP1B1, EZR, SVIP |
| GO:0004180~carboxypeptidase activity | 5 | 0.001083515 | CTSA, CPE, CPXM1, PRCP, AEBP1 |
| GO:0005515~protein binding | 279 | 0.001669375 | AHCYL1, SPARC, TRAF3IP3, PLEKHB2, HHIP, ANTXR1, CCAR2, AQP1, CRKL, SOX2, HERC5, GJA1, RPS16, EDNRB, CDC23, CREB3L3, TMEM88, PPP4R4, DPYSL3, ANKFY1, SOX9, SOX6, SCN1B, WSB1, DNAAF2, ANK2, ACTN4, DICER1, PRAP1, ATG12, EREG, WDR77, AR, PPP1R3C, EWSR1, RFX4, MAP1A, PSME4, RRAGD, ZNF711, TLN2, PRKD1, EZR, UBA52, ALDH7A1, KCTD15, PFN2, SKAP2, GPM6A, SPTBN4, ADCYAP1R1, SDC4, SDC2, TSHZ2, MST1R, KIAA0040, APH1A, DYNC2LI1, MAATS1, FLRT3, ZNHIT6, PRDX1, TSPAN6, PCBP1, SWI5, SARAF, S100A10, CTC1, NDFIP1, HOMER2, NEBL, RDX, RAB3IP, EIF2AK3, NR2F1, DDIT4L, PARVA, ERLIN2, PA2G4, GNG12, PAICS, PBX1, LRP2BP, HNRNPF, CDC16, ID4, ALDH18A1, CD24, PAM, CD200, GSTM5, DAZAP2, GFM1, SLC44A3, RPLP1, CETN2, SMG5, TMSB4X, BBS7, EMD, TMED5, HAVCR2, BRD2, SHPRH, MYBPC1, PGAM1, CCDC113, NME3, ANO6, PROX1, MTSS1, MRPL43, C17ORF82, F5, HADHB, ANO1, MMP14, GJD3, MDFIC, GAN, CDC42EP4, PMP2, ARHGEF1, CD47, MICU1, PLCB1, CRELD2, CACUL1, CD44, YAP1, KANK2, TOMM40, AHNAK, RPL11, ALOX12B, AFP, PPP2CB, CHN1, RPL14, CKB, RPS27A, CD55, IL33, GOLM1, SORT1, AKR1C1, LHFPL5, BBOX1, GPR1, MYO9B, C1ORF21, SVIP, NUDT21, TTC8, FABP5, MYBBP1A, FAS, NCAPD2, TJP2, KCNG3, COL16A1, ICE2, WWC1, CTNND2, NAB2, C4ORF19, JADE2, RPL10A, FGF1, CLU, LAPTM4B, AKR7A2, ARHGDIA, PIM1, NBN, SLC39A7, NUDT15, KPNA1, LMO3, APLP2, TPM1, RPS6, SPICE1, THOC1, CASK, ZBTB33, FOXP4, ATP1B1, PYGO1, DOK3, MAPKAPK2, NOTCH2, SEMA7A, NTM, ARHGAP17, CSF2RB, NDRG2, ZMAT3, UBC, LONRF1, SMAD1, CCDC137, WFS1, CCDC14, CAV1, CYBRD1, MCC, LSM5, ADAP1, HOPX, LSM3, AZIN1, TMEM231, ARHGAP32, ARPC2, MKRN2, ALPL, CYCS, TCF3, CNOT8, BMPR1B, HSPB8, FHL1, SYNE4, GPHN, CDC73, UFL1, SIN3A, IQUB, SCRN2, USP46, SUN2, RBM17, ACTR5, BCKDHB, PEX2, NAV2, PLRG1, POMGNT1, TGFBR3, SLC25A18, RCN1, RCN2, MEP1B, EEF1D, RPL37A, BIRC6, NDUFS1, BIRC7, PRPS1, ROCK1, BRAT1, LENG8, PRCP, PBXIP1, TOB1, CLN5, ABLIM1, POLD2, RAB29, CDC37L1, SH3BP4, LRRC8D, RGS20, MAPK1, PDLIM5, UGGT2, MTA3, CYTH1, LGI1, EYA2, TAF13, PDE4DIP, HNRNPAB, FAM167A, WEE1, VPS45, CDR2L, PFKM |
| GO:0004871~signal transducer activity | 14 | 0.005051335 | NDFIP1, GNG12, CRKL, GJA1, PLCXD3, GNAL, PLCZ1, EEF1D, TSPAN6, MAPKAPK2, FAS, LGALS9, CD24, PLCB1 |
| GO:0044822~poly(A) RNA binding | 46 | 0.010628989 | GFM1, AHNAK, CCDC124, RPL11, RPL10A, HTATSF1, RPL8, CCAR2, CRKL, HERC5, RPS16, ZMAT3, TMSB4X, PCBP1, PRDX1, UBC, RPL14, HIST1H1D, RPS27A, DCAF13, CCDC137, RDX, RPS6, SAMD4B, ACTN4, PA2G4, PUS7, HNRNPAB, MRPL43, LSM3, HADHB, DHX40, NUDT21, MYBBP1A, EWSR1, CDC42EP4, HNRNPF, RPL37A, MKRN2, ARHGEF1, ALDH18A1, FAU, EZR, GRSF1, PPIL4, ZNF598 |
| GO:0003779~actin binding | 16 | 0.012132956 | SPTBN4, MYBPC1, HOMER2, TPM1, RDX, MYO9B, PARVA, FMN2, ACTN4, ADD3, IMPACT, ABLIM1, TLN2, EZR, PDLIM5, EMD |
| GO:0004032~alditol:NADP+ 1-oxidoreductase activity | 3 | 0.014985376 | AKR7A2, AKR1C1, AKR1C3 |
| GO:0005516~calmodulin binding | 12 | 0.017728108 | KCNH5, EWSR1, WFS1, MAPKAPK2, CASK, PHKA1, MYO9B, AEBP1, KCNN3, PLCB1, ADD3, RYR3 |
| GO:0005089~Rho guanyl-nucleotide exchange factor activity | 7 | 0.020868758 | PREX2, PLEKHG3, FARP1, ARHGEF9, FGD6, ARHGEF26, ARHGEF1 |
| GO:0016504~peptidase activator activity | 3 | 0.030376873 | MMP14, CAV1, PSME4 |
| GO:0042802~identical protein binding | 31 | 0.031954217 | DAZAP2, PRPS1, AHCYL1, PON2, HSPB8, NAB2, ZNHIT6, PRDX1, MAPK1, SH3BP4, GSTM3, SUN2, SMAD1, APLP2, CAV1, EIF2AK3, BBOX1, PAICS, MTSS1, ETNPPL, NUDT21, EWSR1, PCDHB6, VWA1, FAS, ALDH18A1, PRKD1, MICU1, PFKM, HCN2, GSTM5 |
| GO:0045499~chemorepellent activity | 4 | 0.038858459 | SEMA7A, NRG3, FLRT3, SEMA6D |
| GO:0004181~metallocarboxypeptidase activity | 4 | 0.038858459 | CPXM2, CPE, CPXM1, AEBP1 |
| GO:0005227~calcium activated cation channel activity | 3 | 0.049829242 | ANO1, ANO6, TRPM3 |
| GO:0044548~S100 protein binding | 3 | 0.049829242 | AHNAK, FGF1, EZR |
