# Supplementary material for: Identification of Novel Key Genes and Pathways in Multiple Sclerosis Based on Weighted Gene Coexpression Network Analysis and Long Noncoding RNA-Associated Competing Endogenous RNA Network
Source: Oxid Med Cell Longev. 2022 Mar 2;2022:9328160. doi: 10.1155/2022/9328160 (PMC8915924; doi:10.1155/2022/9328160)
Supplement: Supplementary 2 — Supplementary Table 2: KEGG analysis of genes in cyan module. [file 9328160.f2.docx]

**Supplementary Table2. KEGG analysis of genes in cyan module**

| **Term** | **Count** | **PValue** | **Genes** |
| --- | --- | --- | --- |
| hsa03010:Ribosome | 16 | 2.74E-05 | RPLP1, MRPL19, RPS6, RPL11, RPL10A, RPL8, RPS16, RPS29, RPL37A, RPL14, RPL38, FAU, RPS27A, RPL18, UBA52, RPL39 |
| hsa04713:Circadian entrainment | 8 | 0.03262712 | ADCYAP1R1, MAPK1, ADCY2, ADCY8, GNG12, PLCB1, RYR3, CACNA1G |
| hsa04261:Adrenergic signaling in cardiomyocytes | 10 | 0.033202664 | PPP2CB, CACNB4, CREB3L3, TPM1, MAPK1, ADCY2, ATP1B1, ADCY8, PLCB1, SCN1B |
| hsa01130:Biosynthesis of antibiotics | 13 | 0.039930476 | PRPS1, PGAM1, AK1, BCKDHB, NME3, PAICS, HADHB, ALDH2, CAT, OGDH, RGN, ALDH7A1, PFKM |
