# Supplementary material for: Identification of Novel Key Genes and Pathways in Multiple Sclerosis Based on Weighted Gene Coexpression Network Analysis and Long Noncoding RNA-Associated Competing Endogenous RNA Network
Source: Oxid Med Cell Longev. 2022 Mar 2;2022:9328160. doi: 10.1155/2022/9328160 (PMC8915924; doi:10.1155/2022/9328160)
Supplement: Supplementary 3 — Supplementary Table 3: the genes with MCODE score higher than 0.4. [file 9328160.f3.docx]

**Supplementary Table.3 The genes with MCODE score higher than 0.4**

| **Name** | **MCODE_Score** |
| --- | --- |
| CACUL1 | 2 |
| ACOX1 | 6 |
| CAT | 6 |
| RPS27A | 14.88970588 |
| KANK2 | 2 |
| ELAVL3 | 2 |
| UBA52 | 14.88970588 |
| UBC | 15 |
| PEX2 | 6 |
| AMACR | 6 |
| HADHB | 1.666666667 |
| ACP5 | 0.5 |
| MAPK1 | 1.7 |
| CKB | 2 |
| ALPL | 1.076923077 |
| FABP5 | 3 |
| LIPC | 2 |
| ALDH18A1 | 3 |
| ACTN4 | 4 |
| SPARC | 4 |
| PUS7 | 2.4 |
| SUN2 | 2 |
| CDC73 | 4 |
| AR | 2.4 |
| AHNAK | 1.2 |
| ARHGDIA | 3 |
| F5 | 9 |
| TMSB4X | 4 |
| CLU | 4 |
| CRKL | 2 |
| TPM1 | 2 |
| EZR | 3.733333333 |
| CETN2 | 3 |
| ADAD1 | 0.5 |
| BBS7 | 1.066666667 |
| PLCZ1 | 0.666666667 |
| ADAP1 | 0.666666667 |
| LMF1 | 0.666666667 |
| ADCY2 | 4 |
| NME3 | 1.2 |
| ADCY8 | 4 |
| GNAL | 3 |
| PLCB1 | 3 |
| ALDH7A1 | 1.2 |
| TAS2R19 | 4 |
| GNG12 | 4 |
| CNR1 | 4 |
| ADCYAP1R1 | 3 |
| WFS1 | 9 |
| CASK | 1.666666667 |
| NTRK2 | 1.2 |
| RFX4 | 0.666666667 |
| ADD3 | 0.666666667 |
| DCLK1 | 2 |
| PLCXD3 | 0.5 |
| TCF3 | 1.2 |
| AFP | 9 |
| RCN1 | 9 |
| SOX9 | 1.230769231 |
| GSTM3 | 3 |
| APLP2 | 9 |
| TNC | 9 |
| SDC2 | 9 |
| SOX2 | 3.466666667 |
| PIGC | 0.5 |
| GOLM1 | 9 |
| CD24 | 3.238095238 |
| CD44 | 1.815789474 |
| VWA1 | 9 |
| SPARCL1 | 9 |
| HNRNPAB | 4 |
| S100A10 | 0.666666667 |
| EEF1D | 14 |
| FAU | 14.88970588 |
| AK1 | 0.5 |
| PFKM | 1.666666667 |
| AKR1C1 | 1.4 |
| AKR7A2 | 1.666666667 |
| AKR1C3 | 2 |
| EPHX1 | 3 |
| ETV2 | 0.666666667 |
| DDIT4L | 0.5 |
| ALDH5A1 | 3 |
| OGDH | 1.857142857 |
| NUDT15 | 0.666666667 |
| ALDH2 | 2 |
| AZIN1 | 2 |
| CDC23 | 9 |
| AQP4 | 0.722222222 |
| GFM1 | 13 |
| PAICS | 4 |
| PRPS1 | 1.4 |
| HMGCLL1 | 2 |
| NFIB | 2 |
| TOMM40 | 1.2 |
| RDX | 4 |
| NDUFS1 | 3 |
| CYP2J2 | 0.666666667 |
| CD109 | 3 |
| CYCS | 6 |
| NRN1 | 3 |
| COL4A6 | 3 |
| NTM | 3 |
| ANGEL1 | 2 |
| RPS16 | 14.88970588 |
| RPS29 | 14.88970588 |
| CNOT8 | 0.666666667 |
| ANK2 | 1.714285714 |
| SPTBN4 | 1.666666667 |
| GJA1 | 2.142857143 |
| MAP1A | 0.5 |
| CNTN1 | 0.952380952 |
| CD55 | 0.952380952 |
| SCN1B | 1.066666667 |
| ANKFY1 | 0.666666667 |
| CTNND2 | 2 |
| FIBIN | 0.5 |
| ANO1 | 2 |
| GABRB1 | 1.2 |
| MTCH2 | 0.952380952 |
| ANO6 | 1.2 |
| SVIP | 2 |
| CD47 | 2.7 |
| CAV1 | 2.4 |
| ARHGAP32 | 1.666666667 |
| UGGT2 | 0.666666667 |
| APH1A | 2 |
| NOTCH2 | 1.4 |
| TSPAN6 | 0.5 |
| RYR3 | 0.5 |
| CD200 | 3 |
| PMP2 | 1.666666667 |
| GPM6A | 2 |
| LGI1 | 0.666666667 |
| SLC39A12 | 2 |
| SLCO1C1 | 2 |
| WDR77 | 2 |
| PRDX1 | 2.4 |
| CMTM2 | 0.666666667 |
| PA2G4 | 14 |
| CCAR2 | 0.666666667 |
| PIM1 | 0.666666667 |
| SMAD1 | 1.607142857 |
| LSM5 | 8 |
| HAMP | 1.2 |
| MYBBP1A | 2.4 |
| BRD2 | 0.5 |
| YAP1 | 1.472727273 |
| RHOQ | 1.066666667 |
| ARPC2 | 3 |
| CDC42EP4 | 2 |
| ARHGEF1 | 0.785714286 |
| ROCK1 | 2.7 |
| FAS | 3 |
| SPICE1 | 2 |
| GLIS3 | 1.2 |
| ARHGEF9 | 1.666666667 |
| NRXN3 | 1.2 |
| GPHN | 1.666666667 |
| ARRDC2 | 0.5 |
| HECW1 | 3.733333333 |
| CPXM1 | 0.666666667 |
| ASTN2 | 2 |
| CDH10 | 2 |
| ATG12 | 4 |
| NDUFV1 | 2.2 |
| NDRG2 | 0.666666667 |
| B3GAT2 | 4 |
| GPC6 | 3.428571429 |
| SDC4 | 2.777777778 |
| GPC4 | 3.428571429 |
| USP39 | 8 |
| IFT22 | 2 |
| DYNC2LI1 | 2 |
| RAB3IP | 2 |
| TTC8 | 1.666666667 |
| BCKDHB | 0.5 |
| BIRC6 | 0.666666667 |
| BIRC7 | 2 |
| BMPR1B | 0.5 |
| TGFBR3 | 4 |
| SIN3A | 2 |
| C12orf66 | 2 |
| RPL8 | 14.88970588 |
| RPL11 | 14.88970588 |
| CACNA1G | 1.2 |
| KCNN3 | 2 |
| CACNB4 | 2 |
| PPP2CB | 5 |
| KBTBD11 | 0.666666667 |
| SETD4 | 0.666666667 |
| RGN | 0.666666667 |
| MMP14 | 3 |
| SLC16A4 | 0.666666667 |
| EDNRB | 3 |
| PPIL4 | 8 |
| CCDC137 | 3 |
| RPL38 | 14.88970588 |
| RPL37A | 14.88970588 |
| IQUB | 0.666666667 |
| CCDC80 | 0.666666667 |
| NAP1L3 | 0.5 |
| TRIL | 0.5 |
| TNFSF4 | 0.5 |
| HAVCR2 | 1.666666667 |
| CD99 | 0.666666667 |
| LGALS9 | 2 |
| FGF1 | 4 |
| SKAP2 | 0.666666667 |
| PGAP3 | 0.5 |
| CFB | 1.4 |
| EWSR1 | 3 |
| CDC16 | 9 |
| WSB1 | 9 |
| HERC5 | 9 |
| LONRF1 | 9 |
| UFL1 | 9 |
| PSME4 | 4.761904762 |
| GAN | 9 |
| DNAJC9 | 2 |
| USP46 | 4 |
| CDH24 | 0.666666667 |
| FRAS1 | 0.666666667 |
| CMPK1 | 0.666666667 |
| CMTM4 | 0.666666667 |
| DPY19L3 | 0.5 |
| TOB1 | 2 |
| CREB3L3 | 2 |
| LHFPL5 | 0.666666667 |
| COL16A1 | 1.666666667 |
| PLOD2 | 2 |
| PAM | 0.666666667 |
| PBX1 | 2 |
| PRKD1 | 0.5 |
| CSF2RB | 0.666666667 |
| LRIG1 | 1.714285714 |
| ZBTB33 | 0.666666667 |
| CTSA | 2.2 |
| RCN2 | 0.5 |
| PRCP | 2 |
| CYBRD1 | 2 |
| HEPH | 1.2 |
| PPP4R4 | 2 |
| EIF2AK3 | 2 |
| RPL10A | 14.88970588 |
| CYP2F1 | 3 |
| GSTM5 | 3 |
| DAZAP2 | 0.666666667 |
| SOX6 | 0.666666667 |
| DCAF13 | 6 |
| DDX59 | 2 |
| PLRG1 | 8 |
| RPP14 | 6 |
| RPS6 | 14.88970588 |
| HOPX | 3 |
| DHX40 | 0.5 |
| TMEM231 | 0.666666667 |
| DICER1 | 4 |
| RNASEH2A | 2 |
| MRPL43 | 7 |
| MRPL19 | 6.805555556 |
| RSPH6A | 0.5 |
| DOK5 | 0.666666667 |
| LPAR4 | 3 |
| PCBP1 | 8 |
| RPLP1 | 14.88970588 |
| RPL39 | 14.88970588 |
| RPL14 | 14.88970588 |
| RPL18 | 14.88970588 |
| EMD | 2 |
| SYNE4 | 2 |
| FHL1 | 0.5 |
| TJP2 | 2 |
| EREG | 0.666666667 |
| ERLIN2 | 4 |
| NUDT21 | 8 |
| LSM3 | 8 |
| HTATSF1 | 0.5 |
| HNRNPF | 8 |
| NBN | 4 |
| FAM167A | 0.666666667 |
| SH3BGR | 0.666666667 |
| KCNF1 | 12 |
| KCNG3 | 12 |
| SMG5 | 15 |
| FGD6 | 0.5 |
| USP16 | 3 |
| RRAGD | 0.5 |
| FLRT3 | 2 |
| FGFRL1 | 2 |
| MYBPC1 | 0.5 |
| NFIA | 2 |
| FMN2 | 0.666666667 |
| PROX1 | 0.5 |
| GOLIM4 | 0.666666667 |
| GPR6 | 0.666666667 |
| GYG2 | 4 |
| PPP1R3C | 4 |
| VTCN1 | 0.666666667 |
| KCNK2 | 0.5 |
| SHPRH | 4 |
| HIST1H1D | 2 |
| RBM17 | 8 |
| SLC39A7 | 0.5 |
| ICE2 | 0.666666667 |
| TAF13 | 0.666666667 |
| ID4 | 1.666666667 |
| POU3F1 | 2 |
| IL33 | 0.666666667 |
| ITGBL1 | 0.666666667 |
| RAB30 | 0.5 |
| SLITRK5 | 0.5 |
| KCTD15 | 2 |
| STRIP2 | 0.5 |
| LRRC8D | 0.666666667 |
| MAPKAPK2 | 2 |
| MEP1B | 0.666666667 |
| MGAT2 | 0.666666667 |
| SLC35C1 | 0.666666667 |
| MST1R | 0.666666667 |
| SEMA6D | 0.666666667 |
| NR2F1 | 0.5 |
| POLD2 | 4 |
| PBXIP1 | 0.666666667 |
| PHKA1 | 0.666666667 |
| SPRED3 | 4 |
| RAB29 | 0.666666667 |
| WWC1 | 2 |
| RNFT1 | 2 |
