# Supplementary material for: Identification of Novel Key Genes and Pathways in Multiple Sclerosis Based on Weighted Gene Coexpression Network Analysis and Long Noncoding RNA-Associated Competing Endogenous RNA Network
Source: Oxid Med Cell Longev. 2022 Mar 2;2022:9328160. doi: 10.1155/2022/9328160 (PMC8915924; doi:10.1155/2022/9328160)
Supplement: Supplementary 4 — Supplementary Table 4: GO analysis of mRNA targets in ceRNA network. [file 9328160.f4.docx]

**Supplementary Table4. GO analysis of mRNA targets in ceRNA network**

| **Term** | **Count** | **PValue** | **Genes** |
| --- | --- | --- | --- |
| GO:0030509~BMP signaling pathway | 4 | 9.65E-04 | TGFBR3, SMAD1, BMPR1B, TOB1 |
| GO:0048812~neuron projection morphogenesis | 3 | 0.006705 | GJA1, WEE1, DICER1 |
| GO:0050680~negative regulation of epithelial cell proliferation | 3 | 0.009037 | TGFBR3, MCC, MTSS1 |
| GO:0045669~positive regulation of osteoblast differentiation | 3 | 0.01032 | SMAD1, GJA1, BMPR1B |
| GO:0030335~positive regulation of cell migration | 4 | 0.011583 | SUN2, SEMA7A, SEMA6D, MAPK1 |
| GO:0008543~fibroblast growth factor receptor signaling pathway | 3 | 0.018688 | HNRNPF, MAPK1, FGFRL1 |
| GO:0042698~ovulation cycle | 2 | 0.020307 | GABRB1, BMPR1B |
| GO:0060044~negative regulation of cardiac muscle cell proliferation | 2 | 0.025321 | GJA1, KCNK2 |
| GO:0006468~protein phosphorylation | 5 | 0.028772 | ROCK1, PIM1, MAPK1, PHKA1, BMPR1B |
| GO:2000279~negative regulation of DNA biosynthetic process | 2 | 0.03031 | GJA1, KCNK2 |
| GO:0060038~cardiac muscle cell proliferation | 2 | 0.032794 | TGFBR3, SMAD1 |
| GO:0061036~positive regulation of cartilage development | 2 | 0.040212 | SMAD1, BMPR1B |
| GO:0005829~cytosol | 15 | 0.010824 | SMAD1, ROCK1, PHKA1, DICER1, ADD3, CRKL, PPP2CB, GJA1, RRAGD, CMPK1, ANKFY1, MAPK1, LONRF1, PDLIM5, RHOQ |
| GO:0005886~plasma membrane | 17 | 0.013264 | NOTCH2, GABRB1, SEMA7A, ROCK1, SEMA6D, CYBRD1, PHKA1, GNG12, MCC, ADD3, FGFRL1, PREX2, GJA1, PIM1, BMPR1B, KCNK2, RHOQ |
| GO:0001726~ruffle | 3 | 0.017456 | ARHGEF26, ROCK1, MTSS1 |
| GO:0070062~extracellular exosome | 13 | 0.017943 | CTSA, UXS1, CYBRD1, GNG12, CRKL, TGFBR3, PPP2CB, GJA1, CMPK1, ANKFY1, MAPK1, ZNF711, RHOQ |
| GO:0043235~receptor complex | 3 | 0.033105 | NOTCH2, TGFBR3, BMPR1B |
| GO:0030660~Golgi-associated vesicle membrane | 2 | 0.046222 | GJA1, RHOQ |
| GO:0046332~SMAD binding | 3 | 0.005602 | TGFBR3, BMPR1B, TOB1 |
| GO:0030165~PDZ domain binding | 3 | 0.02113 | TGFBR3, GJA1, GNG12 |
| GO:0005515~protein binding | 31 | 0.022065 | NOTCH2, SEMA7A, ROCK1, TOB1, CRKL, PPP2CB, GJA1, ZMAT3, PIM1, ANKFY1, MAPK1, LONRF1, PDLIM5, TMED5, USP46, SUN2, SMAD1, CCDC113, CYBRD1, DICER1, MCC, FOXP4, GNG12, MTSS1, TGFBR3, WEE1, HNRNPF, RRAGD, ZNF711, BMPR1B, KCTD15 |
