# Supplementary material for: Identification of Novel Key Genes and Pathways in Multiple Sclerosis Based on Weighted Gene Coexpression Network Analysis and Long Noncoding RNA-Associated Competing Endogenous RNA Network
Source: Oxid Med Cell Longev. 2022 Mar 2;2022:9328160. doi: 10.1155/2022/9328160 (PMC8915924; doi:10.1155/2022/9328160)
Supplement: Supplementary 5 — Supplementary Table 5: KEGG analysis of mRNA targets in ceRNA network. [file 9328160.f5.docx]

**Supplementary Table5. KEGG analysis of mRNA targets in ceRNA network**

| **Term** | **Count** | **PValue** | **Genes** |
| --- | --- | --- | --- |
| hsa04350:TGF-beta signaling pathway | 5 | 2.15E-04 | SMAD1, PPP2CB, ROCK1, MAPK1, BMPR1B |
| hsa04360:Axon guidance | 4 | 0.010506 | SEMA7A, ROCK1, SEMA6D, MAPK1 |
| hsa04910:Insulin signaling pathway | 4 | 0.013155 | MAPK1, PHKA1, RHOQ, CRKL |
| hsa05206:MicroRNAs in cancer | 5 | 0.018561 | NOTCH2, ROCK1, PIM1, DICER1, CRKL |
| hsa05131:Shigellosis | 3 | 0.022278 | ROCK1, MAPK1, CRKL |
| hsa04062:Chemokine signaling pathway | 4 | 0.028896 | ROCK1, MAPK1, GNG12, CRKL |
| hsa04810:Regulation of actin cytoskeleton | 4 | 0.039356 | ROCK1, MAPK1, GNG12, CRKL |
